# Immunotherapeutic potential of CD4 and CD8 single-positive T cells in thymic epithelial tumors

**DOI:** 10.1038/s41598-020-61053-8

**Published:** 2020-03-04

**Authors:** Yoko Yamamoto, Kota Iwahori, Soichiro Funaki, Mitsunobu Matsumoto, Michinari Hirata, Tetsuya Yoshida, Ryu Kanzaki, Takashi Kanou, Naoko Ose, Masato Minami, Eiichi Sato, Atsushi Kumanogoh, Yasushi Shintani, Meinoshin Okumura, Hisashi Wada

**Affiliations:** 1 Department of General Thoracic Surgery, Osaka University Graduate School of Medicine, Suita, Osaka Japan; 2 Department of Clinical Research in Tumor Immunology, Graduate School of Medicine, Osaka University, Suita, Osaka Japan; 3 Department of Respiratory Medicine and Clinical Immunology, Graduate School of Medicine, Osaka University, Suita, Osaka Japan; 4 Drug Discovery & Disease Research Laboratory, Shionogi & Co., Ltd., Osaka, Japan; 5 Department of Frontier Research in Tumor Immunology, Graduate School of Medicine, Osaka University, Suita, Osaka Japan; 6 Department of Pathology (Medical Research Center), Institute of Medical Science, Tokyo Medical University, Tokyo, Japan; 7 Department of Thoracic Surgery, Toneyama National Hospital, Osaka, Japan

**Keywords:** Tumour immunology, Tumour immunology

## Abstract

Indications for current immune checkpoint inhibitors are expanding and now include thymic epithelial tumors (TETs). Although clinical trials on immune checkpoint inhibitors for TETs are ongoing, a rationale has not yet been established for immunotherapy for TETs. Therefore, we herein performed phenotypic and functional analyses of T cells in surgically resected TET tissues with a focus on the anti-tumor properties of T cells to TETs as a step towards establishing a rationale for immunotherapy for TETs. We examined T-cell profiles in surgically resected TET tissues, particularly CD4 and CD8 single-positive T cells, using flow cytometry. In the functional analysis of T cells in TETs, we investigated not only cytokine production by T cells, but also their cytotoxicity using bispecific T-cell engager technology. The cluster analysis of T-cell profiles based on flow cytometric data revealed that type B3 thymoma and thymic carcinoma (B3/C) belonged to the hot cluster characterized by a high proportion of Tim-3+ and CD103+ in CD4 and CD8 single-positive T cells. Enhancements in cytokine production and the cytotoxicity of T cells by the anti-PD-1 antibody were significantly greater in B3/C. These results indicate the potential of immunotherapy for patients with B3/C.

## Introduction

Most anterior mediastinal tumors are thymic epithelial tumors (TETs) including thymoma and thymic carcinoma, which originate in the thymus. According to the World Health Organization (WHO) histopathological classification, TETs are classified as thymoma (types A, AB, B1, B2, and B3) or thymic carcinoma (type C in the 2004 classification) based on the morphology of epithelial tumor cells and the extent of intratumor lymphocytic involvement^1–3^. The complexity, rarity, and heterogeneity of this disease and the lack of *in vitro* and *in vivo* models make it difficult to develop standard treatments. Complete surgical resection is reportedly the only chance for a cure in TETs^4,5^. However, even after complete resection, the recurrence rates of type B3 and type C thymoma (thymic carcinoma) are 27 and 50%, respectively^6^. Surgery cannot be indicated for some patients when tumors invade the surrounding organs, such as the heart and great vessels, and metastasize to multiple organs. More aggressive histological types of TETs often present at an advanced stage and result in worse overall survival. Chemotherapy, radiation therapy, and molecular-targeting agents are also options in combinatorial treatment strategies^7,8^.

Immune checkpoint inhibitors began a new era in cancer immunotherapy. The anti-PD-1 blocking antibody exerts beneficial effects in a limited population of cancer patients^9^. Indications for the anti-PD-1 blocking antibody are expanding and now include TETs. Clinical trials on immune checkpoint inhibitors are ongoing, and acceptable clinical efficacies of the anti-PD-1 antibody have been reported for TETs^10,11^. In the development of anti-PD-1 therapy for TETs, it is crucial to establish a method that identifies target patients who are more likely to respond to the drug. Therefore, it is important to have a clear understanding of the tumor immune microenvironment of TETs. However, the lack of *in vitro* and *in vivo* models makes it difficult to study the tumor immune microenvironment of TETs. The method currently available for the classification of TETs is the WHO histopathological classification, which is based on the morphology of epithelial tumor cells and the proportion of intratumor lymphocytic involvement. The majority of intratumor lymphocytes of TETs are CD4+CD8+ double-positive T cells, which are undifferentiated and functionally immature T cells. On the other hand, CD4 or CD8 single-positive T cells play major roles in cancer immunology. However, the roles of CD4 and CD8 single-positive T cells in TETs have not yet been elucidated in detail from the aspect of cancer immunology. Therefore, in the present study, we focused on the phenotypic and functional properties of CD4 and CD8 single-positive T cells in surgically resected TETs as a step towards establishing a rationale for immunotherapy for TETs.

## Results

### Clinicopathological findings

The clinical and pathological features of patients with TETs are summarized in Table 1. Thirty-one cases of TETs included 10 males (32%) and 21 females (68%) with a mean age of 58 years old (range: 36–82). Thymic carcinoma included 4 squamous cell carcinomas and 2 lymphoepithelioma-like carcinomas. Four patients had a medical history of myasthenia gravis (MG). Three of these patients were diagnosed with type B1 thymoma, and acetylcholinesterase inhibitors were administered to control MG symptoms. The remainder of patients were diagnosed with type B2 thymoma without medication for MG. One patient diagnosed with type AB thymoma had a medical history of pure red cell aplasia (PRCA), and cyclosporine was administered preoperatively to control anemia.

**Table 1:** Patient characteristics.

All TET cases	31
Sex	
Male	10
Female	21
Age Range	36–82
Mean	58
WHO classification	
A	1
AB	4
B1	11
B2	8
B3	1
C	6
Masaoka Stage	
I	15
II	12
III	3
IV	1

### T-cell profiles and clinical characteristics of TETs

**Figure 1:**
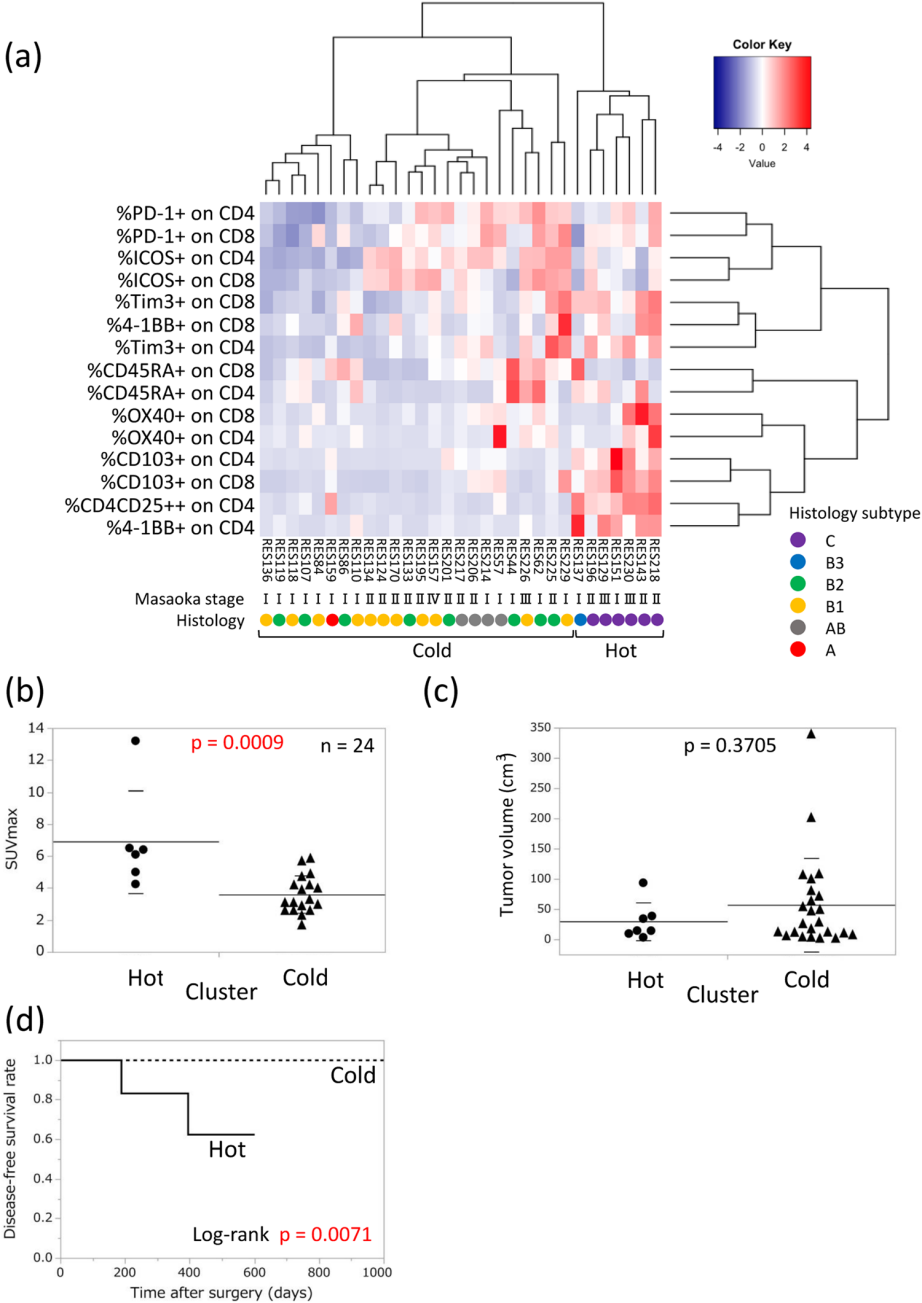
T-cell profiling in TETs. (**a**) Clustering analysis for the T-cell profiling of TETs (n = 31). The T-cell parameters of CD4 and CD8 single-positive T cells measured by flow cytometry are listed. Clinical information shows the pathological WHO histological classification and Masaoka stage. The SUVmax (n = 24) of FDG-PET/CT (**b**) and tumor volumes (**c**) were compared between hot and cold clusters. Tumor volumes were calculated by the following formula: tumor volume (cm^3^) = length × width × thickness/2. Measurements were performed using surgically resected specimens. Means with SD were shown. (**d**) Disease-free survival curve of patients in the hot and cold clusters.

To obtain a clearer understanding of the T-cell immune microenvironment in TETs, we analyzed the T-cell profiles of tumor tissues from surgically resected TETs, with a specific focus on CD4 and CD8 single-positive T cells. Based on flow cytometry data (Supplementary Fig. S1), we performed a cluster analysis of T-cell profiles. We evaluated PD-1 and Tim-3 for T-cell inhibitory molecules and ICOS, 4-1BB, and OX40 for T-cell activation molecules. We also examined CD103 as a marker of tissue resident memory T cells and CD45RA as a T-cell differentiation marker. Correlations were observed between histological types and T-cell profiles. A heat map of TET lymphocytes showed two clearly separated clusters (Fig. 1a). All type B3 thymoma and thymic carcinoma (B3/C) patients belonged to the “hot cluster” and the remainder (non-B3/C) belonged to the “cold cluster”. To elucidate the clinical features of the two separate clusters, we evaluated several clinical factors. Although no significant differences were observed in tumor volumes between the two clusters, the maximum standardized uptake value (SUVmax) of TETs on F-18 fluoro-deoxyglucose-positron emission tomography (FDG-PET)/CT was significantly higher in the hot cluster than in the cold cluster in the 24 patients for whom FDG-PET/CT was performed (Fig. 1b,c). Furthermore, a relationship was observed between T-cell profiles and the Masaoka stage. The weaker expression of immune checkpoint molecules in the cold cluster (non-B3/C) was enriched in Masaoka stage I. We also analyzed disease-free survival (DFS) rates in the hot and cold clusters. The median follow-up time was 14.9 months. DFS rates were significantly different between the hot and cold clusters (p = 0.0071, Fig. 1d). Among molecules expressed on T cells, we found that high expression levels of Tim-3, 4-1BB, CD103, and CD4+CD25++ were related to worse DFS rates (Supplementary Fig. S2). These results revealed a strong correlation between T-cell profiles and clinical characteristics, particularly histological subtypes (Supplementary Table S1). Therefore, we focused on the relationship between T-cell properties and histological subtypes in TETs, comparing B3/C with non-B3/C.

### Expression of immune checkpoint molecules on CD4 and CD8 single-positive T cells in TETs

**Figure 2:**
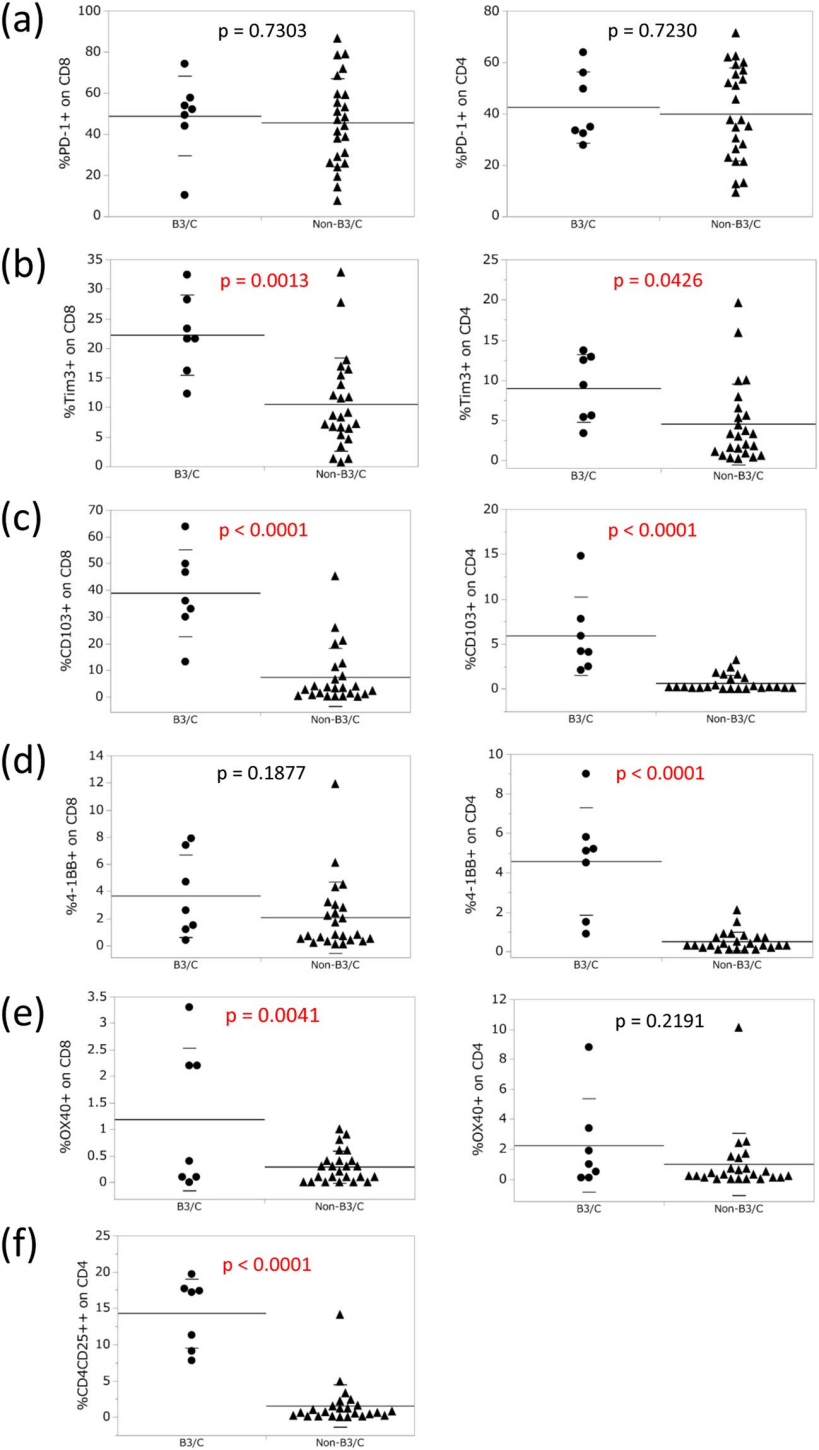
Expression of each immune checkpoint molecule on CD4 and CD8 single-positive T cells in B3/C and non-B3/C TET tissues. The percentages of PD-1 (**a**), Tim-3 (**b**), CD103 (**c**), 4-1BB (**d**), and OX-40 (**e**) expression on CD8 or CD4 single-positive T cells were analyzed by flow cytometry. The ratio of CD4+ CD25++ in CD4 single-positive T cells was also indicated (**f**). Means with SD were shown.

We confirmed the correlation between each immune checkpoint molecule and histological subtype in TETs. Although the rates of PD-1+ in CD8 and CD4 single-positive T cells did not significantly differ between B3/C and non-B3/C (Fig. 2a), the rates of Tim-3+ and CD103+ in CD4 and CD8 single-positive T cells were significantly higher in B3/C than in non-B3/C (Fig. 2b,c). Compared to Tim-3+ T cells, a marked difference was observed in PD-1 + Tim-3+ T cells between B3/C and non-B3/C (Supplementary Fig. S3). The rate of 4-1BB+ in CD4 single-positive T cells was significantly higher in B3/C than in non-B3/C (Fig. 2d). The rate of OX40+ in CD8 single-positive T cells was higher in B3/C than in non-B3/C (Fig. 2e). The rate of CD4+CD25++, namely, regulatory T cells, in CD4 single-positive T cells was also significantly higher in B3/C (Fig. 2f).

The proportion of CD4+CD8+ double-positive T cells was analyzed between B3/C and non-B3/C. As expected, the proportion of CD4+CD8+ double-positive T cells was significantly lower in B3/C than in non-B3/C (Supplementary Fig. S4A). Similarly, the proportions of CD4 and CD8 single-positive T cells were also higher in B3/C than in non-B3/C (Supplementary Fig. S4B,C).

### Cytokine production by CD4 and CD8 single-positive T cells in TETs

**Figure 3:**
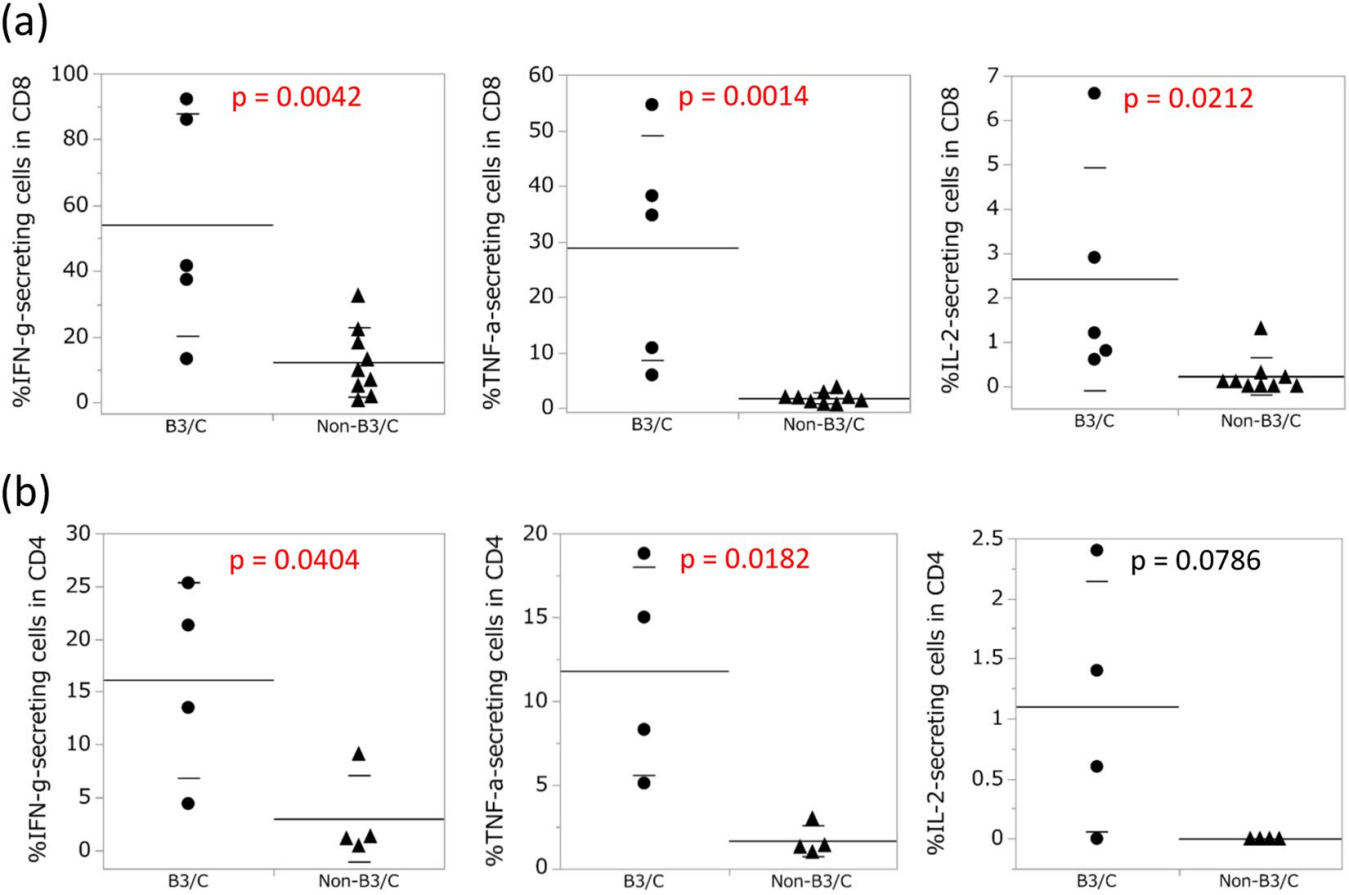
Cytokine production by CD8 and CD4 single-positive T cells in B3/C and non-B3/C TETs with the PMA/ionomycin stimulation. (**a**) Freshly isolated cells from 14 TETs were stimulated with PMA and ionomycin, and the production of IFN-γ, TNF-α, and IL-2 in CD8 single-positive T cells was analyzed by intracellular cytokine staining. Means with SD were shown. (**b**) Purified CD4 single-positive T cells by a cell sorter from eight TETs were stimulated with PMA and ionomycin, and the production of IFN-γ, TNF-α, and IL-2 was analyzed by intracellular cytokine staining. Means with SD were shown.

We evaluated cytokine production by CD4+CD8+ double-positive T cells and CD4 and CD8 single-positive T cells using the PMA/ionomycin stimulation method. IFN-γ production was lower in CD4+CD8+ double-positive T cells than in CD4 and CD8 single-positive T cells (Supplementary Fig. S4D). Moreover, the ability of CD8 single-positive T cells to produce IFN-γ, TNF-α, and IL-2 was significantly greater in B3/C than in non-B3/C (Fig. 3a, Supplementary Fig. S5). Since CD4 expression was down-regulated when CD4 single-positive T cells were activated by the PMA/ionomycin stimulation, we were unable to distinguish activated CD4 single-positive T cells from double-negative lymphocytes in TET lymphocytes. Therefore, before the stimulation, we purified CD4 single-positive T cells using the FACSAria II cell sorter, and found that the ability of CD4 single-positive T cells to produce IFN-γ and TNF-a with the PMA/ionomycin stimulation was also significantly greater in B3/C than in non-B3/C (Fig. 3B, Supplementary Fig. S5).

### Cytotoxicity of T cells in TETs

**Figure 4:**
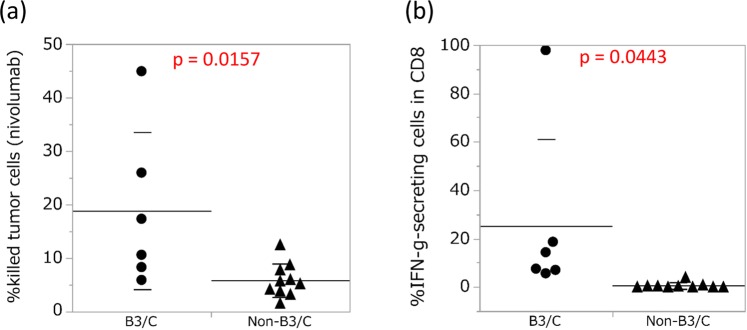
Effects of nivolumab on T-cell cytotoxicity in B3/C and non-B3/C TET tissues. (**a**) The effects of nivolumab for T cells in 16 TET tissues were analyzed by T-cell cytotoxicity using BiTE with nivolumab. (**b**) IFN-γ secretion from CD8 single-positive T cells was also evaluated. Means with SD were shown.

One of the critical factors for immune responses in the tumor microenvironment is the cytotoxicity of tumor antigen-specific T cells. To evaluate the cytotoxicity of T cells in fresh TETs, we developed an assay system using BiTE. In this assay, the cytotoxicity of T cells in freshly isolated cells from TET tissue was analyzed in a co-culture with tumor cells expressing EphA2 (U251) and EphA2/CD3-specific BiTE. To evaluate the effects of nivolumab on T-cell cytotoxicity in TET tissues, we compared T-cell cytotoxicity in TET tissues with the anti-PD-1 antibody (nivolumab) to the IgG4 control using BiTE. We found that the effects of the anti-PD-1 antibody on T-cell cytotoxicity were stronger in B3/C than in non-B3/C (Fig. 4a). We performed an IFN-γ secretion assay to evaluate cytokine production by T cells. Similar to the results obtained for cytotoxicity, IFN-γ secretion from CD8 single-positive T cells was higher in B3/C than in non-B3/C (Fig. 4b, Supplementary Fig. S6). The expression levels of perforin and granzyme B in CD8 single-positive T cells were also higher in B3/C than in non-B3/C (Supplementary Fig. S7). We then evaluated the production of multiple cytokines under the same co-culture conditions. In a quantitative multiplex analysis, the cytokine production of IL-2, IL-4, IL-6, and IFN-γ was significantly greater in B3/C than in non-B3/C (Supplementary Fig. S8). We also performed PD-L1 staining on tumor cells of TET tissues. Although no significant difference was observed in PD-L1 expression by tumor cells between B3/C and non-B3/C, the effects of nivolumab on T-cell cytotoxicity were stronger in patients with high expression levels of PD-L1 in B3/C (Supplementary Table S2 and Supplementary Fig. S9).

Since the proportion of CD8 single-positive T cells in each histological type significantly differed, we purified CD8 single-positive T cells using the FACSAria II cell sorter and evaluated the effects of nivolumab on cytotoxicity. Similar to the bulk results, the effects of nivolumab on the cytotoxicity of CD8 single-positive T cells were slightly stronger in B3/C than in non-B3/C (Supplementary Fig. S10A), and cytokine production levels were also higher in B3/C than in non-B3/C in the IFN-γ secretion assay (Supplementary Fig. S10B) and by the multiplex cytokine analysis (Supplementary Fig. S10C).

The results of these preclinical evaluations indicate that T cells in B3/C are potentially effective as anti-PD-1 therapy.

## Discussion

In the present study, we analyzed T-cell properties in TET tissues from the aspect of anti-tumor immunotherapy, with a specific focus on CD4 and CD8 single-positive T cells. Analyses of T-cell profiles, cytokine production, and T-cell cytotoxicity revealed that the properties of CD4 and CD8 single-positive T cells correlated with the histopathology of TETs in the WHO classification. CD4 and CD8 single-positive T cells showed more favorable characteristics for anti-tumor immunotherapy in B3/C than in non-B3/C according to the WHO classification. Since the WHO classification is based on the histological findings of TET tumors and CD4+CD8+ double-positive T cells, previous studies on TETs mainly focused on CD4+CD8+ double-positive T cells, not CD4 and CD8 single-positive T cells. Although CD4+CD8+ double-positive T cells are critical for T-cell development in the thymus, these T cells are functionally immature and have insufficient anti-tumor activity against TETs^12^. On the other hand, CD4 and CD8 single-positive T cells play crucial roles in anti-tumor immunotherapy, including immune checkpoint inhibitors. However, limited information is currently available on CD4 and CD8 single-positive T cells in TETs for anti-tumor immunotherapy. Previous studies were based on immunohistochemical findings, including PD-1 and PD-L1 staining^13–16^, which limited analyses of the multiple molecular profiles of T cells. Regarding T-cell immune profiles, a flow cytometric analysis provides powerful options^17^. Consistent with previous findings on T-cell profiles in lung cancer tissues^18^, the present results indicated that the CD4 and CD8 single-positive T-cell profiles of TETs in a flow cytometric analysis showed two clearly separated clusters: “hot” and “cold” clusters, which strongly correlated with the WHO classifications of B3/C and non-B3/C. Since thymic epithelial cells induce T-cell differentiation, TET tumors are estimated to form the tumor immune microenvironment of TETs, implying that differences in CD4 and CD8 single-positive T-cell properties are based on differences between B3/C and non-B3/C tumor cells. Although PD-1 expression by T cells did not markedly differ between B3/C and non-B3/C, the rate of PD-1 + Tim-3+ in CD4 and CD8 single-positive T cells was significantly higher in B3/C than in non-B3/C. Based on previous findings^19^, we propose that a specific number of cancer-unrelated T cells exist in PD-1+ T cells. The number of cancer-specific T cells was previously estimated to be higher in PD-1 + Tim-3+ T cells than in PD-1 + T cells. Consistent with previous findings on lung cancer, Tregs strongly infiltrated the TETs of the “hot” cluster^18^. Genetic differences between B3/C and non-B3/C tumor cells have been reported for TETs^20–22^. Significant increases in the tumor mutation burden have been observed in B3/C, specifically thymic carcinoma^21^.

In contrast to the findings of numerous analyses of the tumor properties of TETs, the present study involved experiments that focused on the T-cell properties of TETs, specifically for CD4 and CD8 single-positive T cells. In addition to T-cell profiles, we evaluated T-cell function in TET tissues by means of not only a classical PMA/ionomycin stimulation, but also T-cell cytotoxicity by BiTE. BiTE artificially induces antigen-specific T-cell cytotoxicity, which is partly similar to tumor cell killing activity by endogenous tumor antigen-specific T cells^23,24^. Since BiTE activates T cells via CD3 without a CD28 co-stimulation, it activates effector T cells, which exhibit tumor cell killing activity without a CD28 co-stimulation, but not naïve T-cell clones. Based on these analyses, we found that T cells in B3/C showed a stronger anti-tumor immune response to nivolumab than those in non-B3/C. Clinical trials on immune checkpoint inhibitors for TETs are ongoing, and an acceptable clinical efficacy has been reported for the anti-PD-1 antibody for TETs^10,11^. Similar to TETs, there are currently no appropriate animal models for many types of rare cancers. One of the promising approaches for rare cancers is the evaluation of clinical specimens, specifically tumor tissues. To achieve this, in addition to T-cell profiles and classical T-cell stimulation assays, our method using BiTE represents a useful approach for evaluating antitumor T-cell responses in order to develop anti-tumor immunotherapy for rare tumors such as TETs.

This study had some limitations. The sample size was small because of the rarity of this disease. Furthermore, due to the limited sample size, we were unable to evaluate cytokine production or perform the BiTE assay for type A thymoma.

In conclusion, the characteristics of CD4 and CD8 single-positive T cells in B3 thymoma and thymic carcinoma are favorable for anti-tumor immunity. These results indicate that B3 thymoma and thymic carcinoma are potent targets for anti-tumor immunotherapy.

## Methods

### Patients and tissue samples

Treatment-naïve fresh TETs were surgically resected from 31 patients between August 2015 and September 2018. The present study was approved by the Osaka University Hospital Ethics Committee (control number 13266), and written informed consent was obtained from each patient. A computed tomography (CT) scan was routinely performed every 6 months as patient follow-up. Tumor tissues were minced in a 6-cm dish and digested to a single cell suspension using a Tumor Dissociation Kit for humans (Miltenyi Biotec) and gentle MACS Dissociator (Miltenyi Biotec) according to the manufacturer’s instructions. The cell suspension was applied to a 70-μm nylon cell strainer (BD Biosciences) with the lysis of red blood cells by BD Pharm Lyse. Dead cells and debris were removed by centrifugation in isodensity Percoll solution (Pharmacia Biotech), followed by FACS and T-cell functional analyses.

### Flow cytometric analysis

Surface marker staining was performed after the FcR block using Human TruStain FcX Fc Receptor blocking solution (BioLegend). Cells were incubated with the Live/Dead Fixable Yellow Dead Cell Stain Kit (Life Technologies). Surface marker-stained cells were analyzed on BD LSRFortessa with FACSDiva software (BD Biosciences). The following antibodies were used for FACS staining: anti-CD45RA-FITC (clone HI100), anti-CD25-PE (BC96), anti-4-1BB-BV421 (4B4-1), anti-CD8-BV510 (RPA-T8), anti-CD103-BV605 (Ber-ACT8), anti-CD4-BV711 (OKT4), anti-Tim3-APC, (F38-2E2), anti-CD3-Alexa Fluor 700 (UCHT1), anti-CD45-BV786 (HI30), and IgG1 isotype control (MOPC-21) purchased from Biolegend. Anti-ICOS-PerCP eFluor 710 (ISA-3) and IgG1 isotype control (P3.6.2.8.1) were purchased from eBioscience. Anti-OX-40-PE-CF594 (ACT35), anti-PD-1-PE Cy7 (EH12.1), and IgG1 isotype control (X40) were purchased from BD Bioscience.

### *In vitro* stimulation for intracellular cytokine staining

Freshly isolated cells or CD4 single-positive T cells purified by the FACS Aria II cell sorter (BD Biosciences) from TET tissues were stimulated with 50 ng/ml phorbol 12-myristate 13-acetate (PMA; Sigma-Aldrich), 1 µg/ml ionomycin (Sigma-Aldrich), and Golgi Stop reagent (BD Biosciences) at 37 °C, 5% CO_2_, for 5 hours. Harvested cells were washed and stained with antibodies against surface antigens and fixable viability dye (eBioscience) at 4 °C for 20 minutes. After the incubation, cells were washed, fixed, and permeabilized with Cytofix/Cytoperm solution (BD Bioscience) at 4 °C for 30 minutes. Intracellular cytokines (IFN-γ, TNF-α, and IL-2) were then stained with antibodies for IFN-γ (clone 4SB3; Biolegend), TNF-α (clone MAb11; Biolegend), and IL-2 (clone MQ1-17H12; eBioscience), followed by FACS analyses.

### T-cell cytotoxicity assay

The U251 cell line was kindly provided by Dr. Yasuko Mori (Kobe University, Japan). Cell line authentication by short tandem repeat (STR) profiling and mycoplasma testing was performed at the JCRB Cell Bank. The construction of EphA2/CD3 BiTE containing EphA2-specific scFv 4H5, a short serine-glycine linker, and CD3-specific scFv derived from OKT3 is described elsewhere^25,26^. We previously validated the T-cell cytotoxicity assay^25^. Briefly, U251 cells were plated on 96-well flat-bottomed cell culture plates (Corning) at a density of 1 × 10^4^ cells per well with PRMI medium 1640 (Nacalai Tesque) containing 10% fetal bovine serum (FBS; HyClone, Thermo Scientific). After 24 hours, 5 × 10^4^ freshly isolated cells or CD8 single-positive T cells purified by the FACS Aria II cell sorter (BD Biosciences) from TET tissues were added to plates with 100 ng/ml of EphA2/CD3 BiTE. After a 48-hour co-culture, culture supernatants were cryopreserved, non-adherent cells were removed by gentle washing four times with RPMI medium 1640 containing 10% FBS, and the remaining adherent viable cells were detected by the 3-(4,5-dimethylthiazol-2-yl)-5-(3-carboxymethoxyphenyl)-2-(4-sulfophenyl)-2H-tetrazolium (MTS) assay (CellTiter 96 aqueous one solution cell proliferation assay; Promega). The assay was performed in triplicate. The calculation of EphA2/CD3 BiTE-mediated killing was based on the degree of the reduction in viable target cells with the following formula:

$${\rm{Cytotoxicity}}\,( \% )=[1-({\rm{absorbance}}\,{\rm{of}}\,{\rm{treated}}\,{\rm{wells}})/({\rm{absorbance}}\,{\rm{of}}\,{\rm{non}}-{\rm{treated}}\,{\rm{wells}})]\times 100$$

Each treated well consisted of 1 × 10^4^ U251 cells and 5 × 10^4^ freshly isolated cells from TETs with 100 ng/ml of EphA2/CD3 BiTE. Each non-treated well consisted of 1 × 10^4^ U251 cells and 5 × 10^4^ freshly isolated cells from TETs without EphA2/CD3 BiTE.

In the evaluation of T-cell cytotoxicity enhanced by nivolumab, 1 μg/ml of nivolumab (provided by Ono Pharmaceutical) was added to plates with 100 ng/ml of EphA2/CD3 BiTE. As a control, 1 μg/ml of human IgG4 (Abcam) was added to plates with 100 ng/ml of EphA2/CD3 BiTE. The calculation of % cytotoxicity was based on the degree of the reduction induced in viable target cells by nivolumab using the following formula:

$${\rm{Cytotoxicity}}\,{\rm{by}}\,{\rm{nivolumab}}\,( \% )=[1-({\rm{absorbance}}\,{\rm{of}}\,{\rm{BiTE}}+{\rm{nivolumab}})/({\rm{BiTE}}+{\rm{control}}\,{\rm{IgG4}})]\times 100$$

Collected non-adherent cells were analyzed by the IFN-γ secretion assay (Miltenyi Biotec) according to the manufacturer’s instructions. Briefly, cells were treated with the anti-IFN-γ monoclonal antibody conjugated to a CD45-specific monoclonal antibody (IFN-γ Catch Reagent) on ice for 5 minutes. Cells were then diluted in AIM-V medium and placed on a slow rotating device (Miltenyi Biotec) to allow IFN-γ secretion at 37 °C in a 5% CO_2_ atmosphere. After being incubated for 45 minutes, cells were washed with cold buffer and treated with PE-conjugated anti-IFN-γ (detection reagent) and other T-cell surface markers. Following an incubation at 4 °C for 20 minutes, cells were washed and analyzed by FACS.

Cryopreserved culture supernatants were analyzed by the Bio-Plex Pro^TM^ Human Cytokine Grp I 8-plex Assay (Bio-Rad) according to the manufacturer’s instructions.

The production of each cytokine was calculated by the following formula:

$$\begin{array}{c}{\rm{Cytokine}}\,{\rm{production}}\,({\rm{pg}}/{\rm{ml}})=({\rm{cytokine}}\,{\rm{production}}\,{\rm{of}}\,{\rm{BiTE}}+{\rm{nivolumab}})\\ -\,({\rm{cytokine}}\,{\rm{production}}\,{\rm{of}}\,{\rm{BiTE}}+{\rm{control}}\,{\rm{IgG4}}).\end{array}$$

### Statistical analysis

Data are expressed as the mean ± standard deviation or median values. Differences in clinical variables between groups were evaluated using an unpaired, 2-tailed Student’s *t*-test. In the analysis of recurrence, the starting point was the day of surgery. The end of the DFS period was defined as the day of recurrence. DFS was analyzed by the Kaplan-Meier method with differences between groups being calculated using the Log-rank test. A p value less than 0.05 was considered to be significant. All statistical analyses were performed using JMP Pro 14 software program (SAS Institute). A hierarchical clustering algorithm was applied using Ward’s method by R software. Individual data were transformed to Z scores for standardization purposes.

### Study approval

The present study was conducted according to the principles of the Declaration of Helsinki. The study protocol was approved by the Osaka University Hospital Ethics Committee, and written informed consent was obtained from participants prior to their inclusion in the study.

## Supplementary information

Supplementary Information.

## Data Availability

The authors declare that the main data supporting the results of the present study are available within the article and its Supplementary Information files. Extra data are available from the corresponding author upon request.
