# Supplementary material for: Immunotherapeutic potential of CD4 and CD8 single-positive T cells in thymic epithelial tumors
Source: Sci Rep. 2020 Mar 4;10:4064. doi: 10.1038/s41598-020-61053-8 (PMC7055333; doi:10.1038/s41598-020-61053-8)
Supplement: Supplementary file 1 — Supplementary Information. [file 41598_2020_61053_MOESM1_ESM.pdf]

Immunotherapeutic potential of CD4 and CD8 single-positive T cells in thymic epithelial tumors

Yoko Yamamoto[1,2], Kota Iwahori[2,3*], Soichiro Funaki[1], Mitsunobu Matsumoto[2,4], Michinari Hirata[2,4], Tetsuya Yoshida[4,5], Ryu Kanzaki[1], Takashi Kanou[1], Naoko Ose[1], Masato Minami[1], Eiichi Sato[6], Atsushi Kumanogoh[3], Yasushi Shintani[1], Meinoshin Okumura[7], Hisashi Wada[2]

1 Department of General Thoracic Surgery, Osaka University Graduate School of Medicine, Suita, Osaka, Japan
2 Department of Clinical Research in Tumor Immunology, Graduate School of Medicine, Osaka University, Suita, Osaka, Japan
3 Department of Respiratory Medicine and Clinical Immunology, Graduate School of Medicine, Osaka University, Suita, Osaka, Japan
4 Drug Discovery & Disease Research Laboratory, Shionogi & Co., Ltd., Osaka, Japan
5 Department of Frontier Research in Tumor Immunology, Graduate School of Medicine, Osaka University, Suita, Osaka, Japan
6 Department of Pathology (Medical Research Center), Institute of Medical Science, Tokyo Medical University, Tokyo, Japan
7 Department of Thoracic Surgery, Toneyama National Hospital, Osaka, Japan

*To whom correspondence should be addressed:
Kota Iwahori, MD, PhD
Department of Clinical Research in Tumor Immunology,
Graduate School of Medicine, Osaka University
2-2 Yamadaoka, Suita-shi, Osaka 565-0871, Japan
Tel.: +81-6-6210-8413; Fax: +81-6-6210-8413
E-mail: iwahori@climm.med.osaka-u.ac.jp

Supplementary Methods

Perforin and granzyme B staining

Cryopreserved cells isolated from TET tissues were thawed and 5 × 10^4 cells were co-cultured with 1 × 10^4 U251 cells and 100 ng/ml of EphA2/CD3 BiTE per well. In the evaluation of T-cell cytotoxicity enhanced by nivolumab, 1 μg/ml of nivolumab (provided by Ono Pharmaceutical) was added to plates with 100 ng/ml of EphA2/CD3 BiTE. As a control, 1 μg/ml of human IgG4 (Abcam) was added to plates with 100 ng/ml of EphA2/CD3 BiTE. After a 48-hour co-culture, harvested cells were washed and stained with antibodies against surface antigens and fixable viability dye (eBioscience) at 4 °C for 30 minutes. After the incubation, cells were washed, fixed, and permeabilized with Cytofix/Cytoperm solution (BD Bioscience) at 4 °C for 30 minutes. Perforin and granzyme were then stained with antibodies for perforin (clone B-D48; Biolegend) and granzyme B (clone GB11; Biolegend), followed by FACS analyses.

PD-L1 staining

The rabbit monoclonal anti-human PD-L1 antibody used was clone E1L3N (Cell Signaling). Heat-induced epitope retrieval with High pH Target Retrieval Solution (DAKO) was performed. Endogenous peroxidase activity was blocked by incubating in 0.01M phosphate-buffered saline containing 0.3% hydrogen peroxidase and 0.1% sodium azide, and the EnVision plus system (DAKO) was used for secondary detection. The final product was visualized by 3-3' diaminobenzidine.

Supplementary Figure S1

TIC lymphocytes (RES217)   Type AB thymoma

	FITC	PerCP eFluor 710	PE	PE CF594	PE Cy7	BV421	BV510	BV605	BV711	BV786	APC	Alexa Fluor 700	APC Cy7
TIC lympho	CD45RA	ICOS	CD25	OX40	PD-1	4-1BB	CD8	CD103	CD4	CD45	Tim-3	CD3	Live/Dead
TIC lympho iso	CD45RA	iso	iso	iso	iso	iso	CD8	iso	CD4	CD45	iso	CD3	Live/Dead

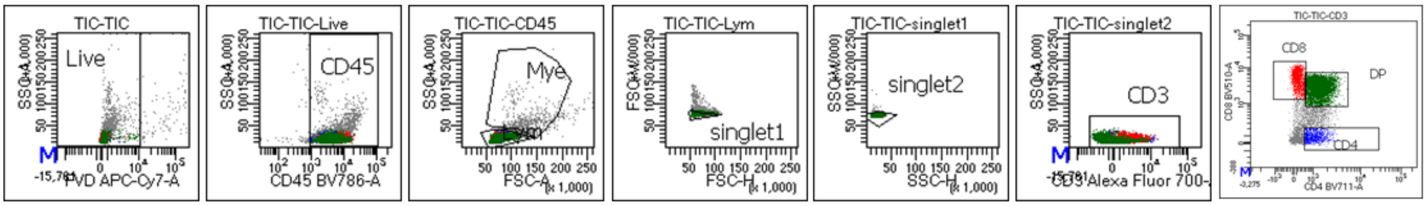

CD4 single-positive T cells

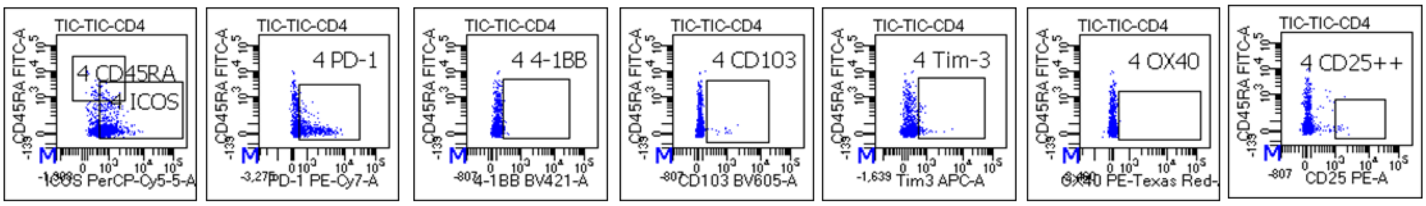

CD8 single-positive T cells

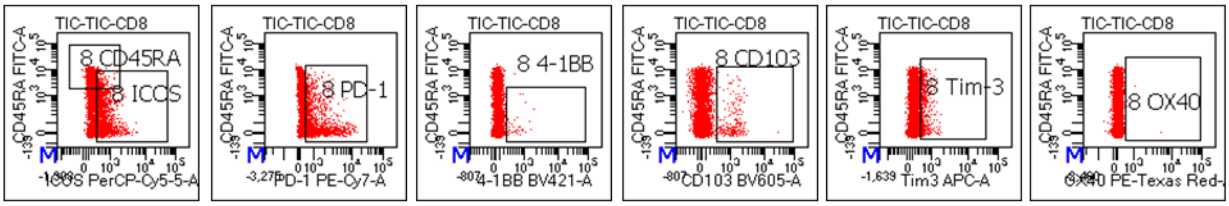

TIC lymphocytes isotype-control (RES217)

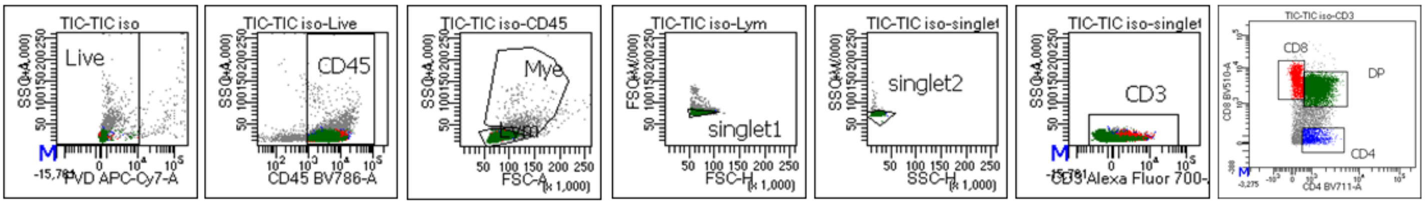

CD4 single-positive T cells isotype-control

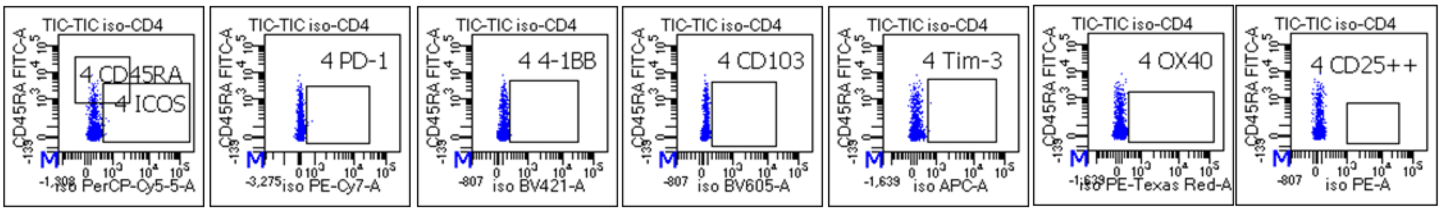

CD8 single-positive T cells isotype-control

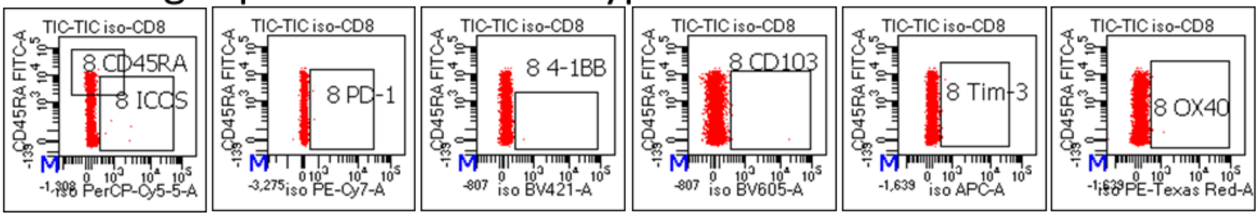

Tube: TIC

Population	#Events	%Parent	%Total
All Events	41,696	####	100.0
Live	41,603	99.8	99.8
CD45	41,523	99.8	99.6
Lym	40,777	98.2	97.8
singlet1	39,762	97.5	95.4
singlet2	39,719	99.9	95.3
CD3	39,716	100.0	95.3
CD4	2,377	6.0	5.7
4 CD25++	15	0.6	0.0
4 CD45RA	77	3.2	0.2
4 ICOS	1,621	68.2	3.9
4 PD-1	532	22.4	1.3
4 4-1BB	14	0.6	0.0
4 CD103	15	0.6	0.0
4 Tim-3	59	2.5	0.1
4 OX40	1	0.0	0.0
CD8	5,542	14.0	13.3
8 CD45RA	1,051	19.0	2.5
8 ICOS	2,208	39.8	5.3
8 PD-1	1,726	31.1	4.1
8 4-1BB	36	0.6	0.1
8 CD103	220	4.0	0.5
8 Tim-3	545	9.8	1.3
8 OX40	8	0.1	0.0
DP	25,724	64.8	61.7
Mye	446	1.1	1.1

Tube: TIC iso

Population	#Events	%Parent	%Total
All Events	48,552	####	100.0
Live	48,406	99.7	99.7
CD45	47,748	98.6	98.3
Lym	46,840	98.1	96.5
singlet1	45,683	97.5	94.1
singlet2	45,631	99.9	94.0
CD3	45,630	100.0	94.0
CD4	3,090	6.8	6.4
4 CD25++	0	0.0	0.0
4 CD45RA	86	2.8	0.2
4 ICOS	8	0.3	0.0
4 PD-1	3	0.1	0.0
4 4-1BB	1	0.0	0.0
4 CD103	0	0.0	0.0
4 Tim-3	4	0.1	0.0
4 OX40	0	0.0	0.0
CD8	7,054	15.5	14.5
8 CD45RA	1,295	18.4	2.7
8 ICOS	4	0.1	0.0
8 PD-1	2	0.0	0.0
8 4-1BB	1	0.0	0.0
8 CD103	5	0.1	0.0
8 Tim-3	5	0.1	0.0
8 OX40	6	0.1	0.0
DP	28,192	61.8	58.1
Mye	563	1.2	1.2

TIC lymphocytes (RES230)   Thymic carcinoma

	FITC	PerCP eFluor 710	PE	PE CF594	PE Cy7	BV421	BV510	BV605	BV711	BV786	APC	Alexa Fluor 700	APC Cy7
TIC lympho	CD45RA	ICOS	CD25	OX40	PD-1	4-1BB	CD8	CD103	CD4	CD45	Tim-3	CD3	Live/Dead
TIC lympho iso	CD45RA	iso	iso	iso	iso	iso	CD8	iso	CD4	CD45	iso	CD3	Live/Dead

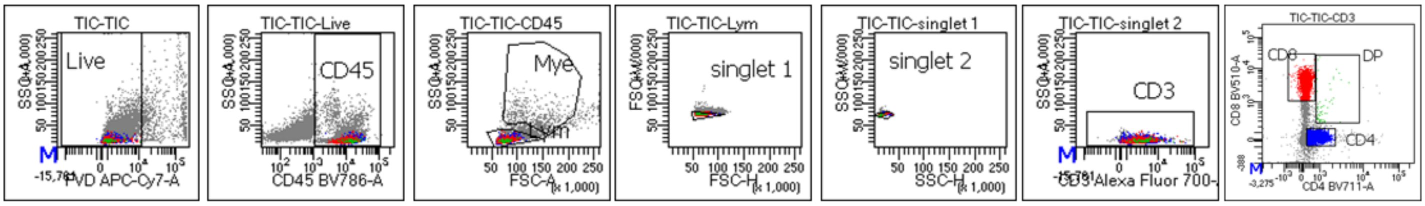

CD4 single-positive T cells

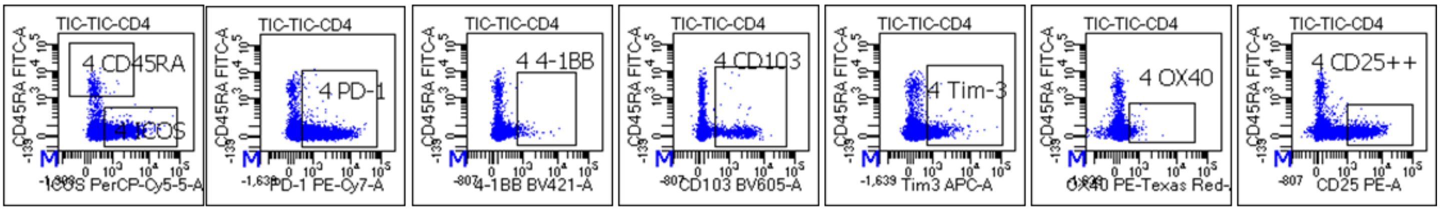

CD8 single-positive T cells

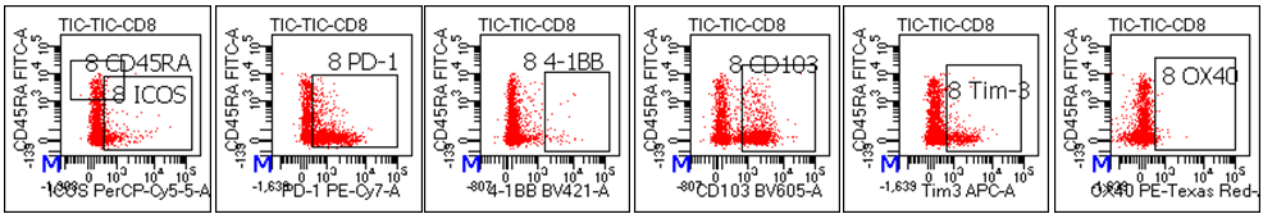

TIC lymphocytes isotype-control (RES230)

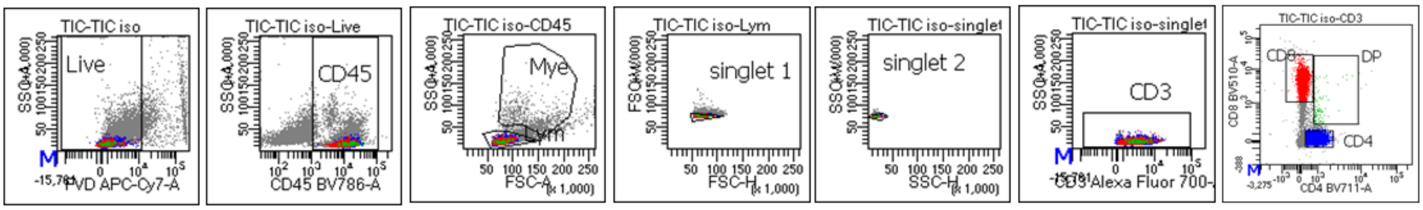

CD4 single-positive T cells isotype-control

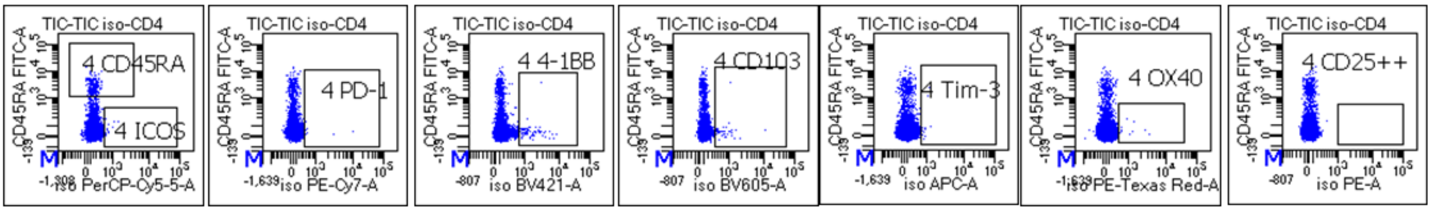

CD8 single-positive T cells isotype-control

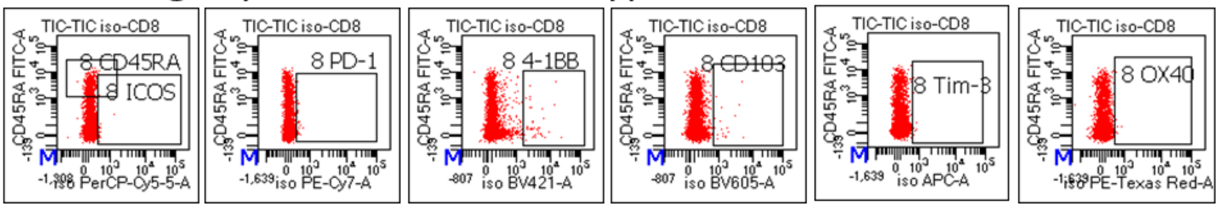

Tube: TIC

Population	#Events	%Parent	%Total
All Events	25,842	####	100.0
Live	25,019	96.8	96.8
CD45	18,927	75.7	73.2
Lym	17,992	95.1	69.6
singlet 1	17,307	96.2	67.0
singlet 2	17,180	99.3	66.5
CD3	17,179	100.0	66.5
CD4	9,981	58.1	38.6
4 CD45RA	197	2.0	0.8
4 CD25++	1,561	15.6	6.0
4 ICOS	3,874	38.8	15.0
4 PD-1	4,286	42.9	16.6
4 4-1BB	90	0.9	0.3
4 CD103	618	6.2	2.4
4 Tim-3	804	8.1	3.1
4 OX40	124	1.2	0.5
CD8	2,971	17.3	11.5
8 CD45RA	238	8.0	0.9
8 PD-1	1,633	55.0	6.3
8 4-1BB	37	1.2	0.1
8 CD103	1,268	42.7	4.9
8 Tim-3	425	14.3	1.6
8 OX40	35	1.2	0.1
8 ICOS	433	14.6	1.7
DP	66	0.4	0.3
Mye	462	2.4	1.8

Tube: TIC iso

Population	#Events	%Parent	%Total
All Events	34,432	####	100.0
Live	33,345	96.8	96.8
CD45	26,277	78.8	76.3
Lym	24,920	94.8	72.4
singlet 1	23,934	96.0	69.5
singlet 2	23,856	99.7	69.3
CD3	23,856	100.0	69.3
CD4	14,934	62.6	43.4
4 CD45RA	242	1.6	0.7
4 CD25++	0	0.0	0.0
4 ICOS	15	0.1	0.0
4 PD-1	8	0.1	0.0
4 4-1BB	77	0.5	0.2
4 CD103	39	0.3	0.1
4 Tim-3	11	0.1	0.0
4 OX40	11	0.1	0.0
CD8	4,528	19.0	13.2
8 CD45RA	455	10.0	1.3
8 PD-1	24	0.5	0.1
8 4-1BB	40	0.9	0.1
8 CD103	22	0.5	0.1
8 Tim-3	9	0.2	0.0
8 OX40	7	0.2	0.0
8 ICOS	20	0.4	0.1
DP	104	0.4	0.3
Mye	686	2.6	2.0

Supplementary Figure S1

Gating strategy of the FACS analysis for T-cell profiles.

The gating strategy of the FACS analysis for tumor-infiltrating cells (TIC) was shown using BD LSRFortessa with FACSDiva software.

Supplementary Figure S2

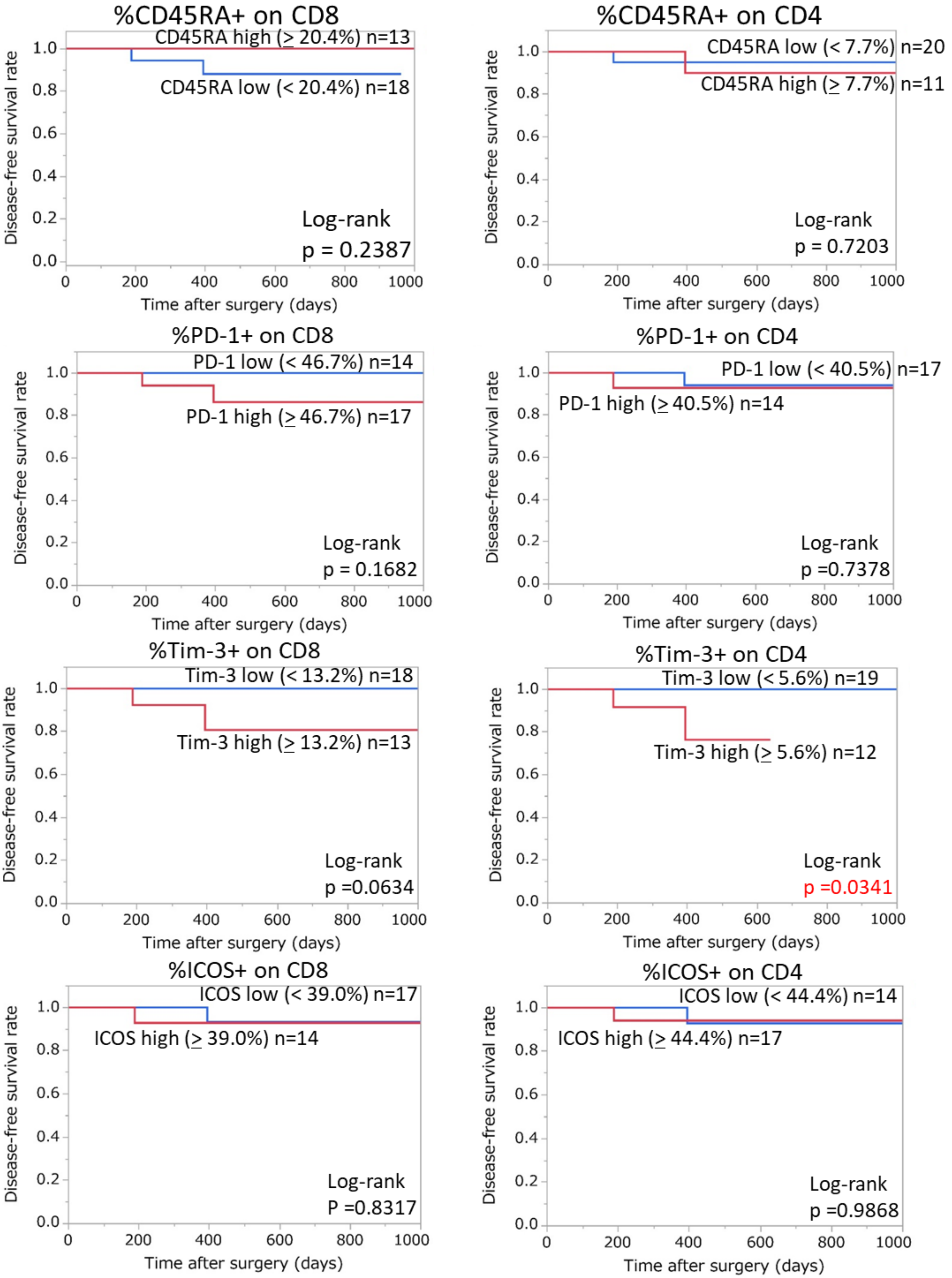

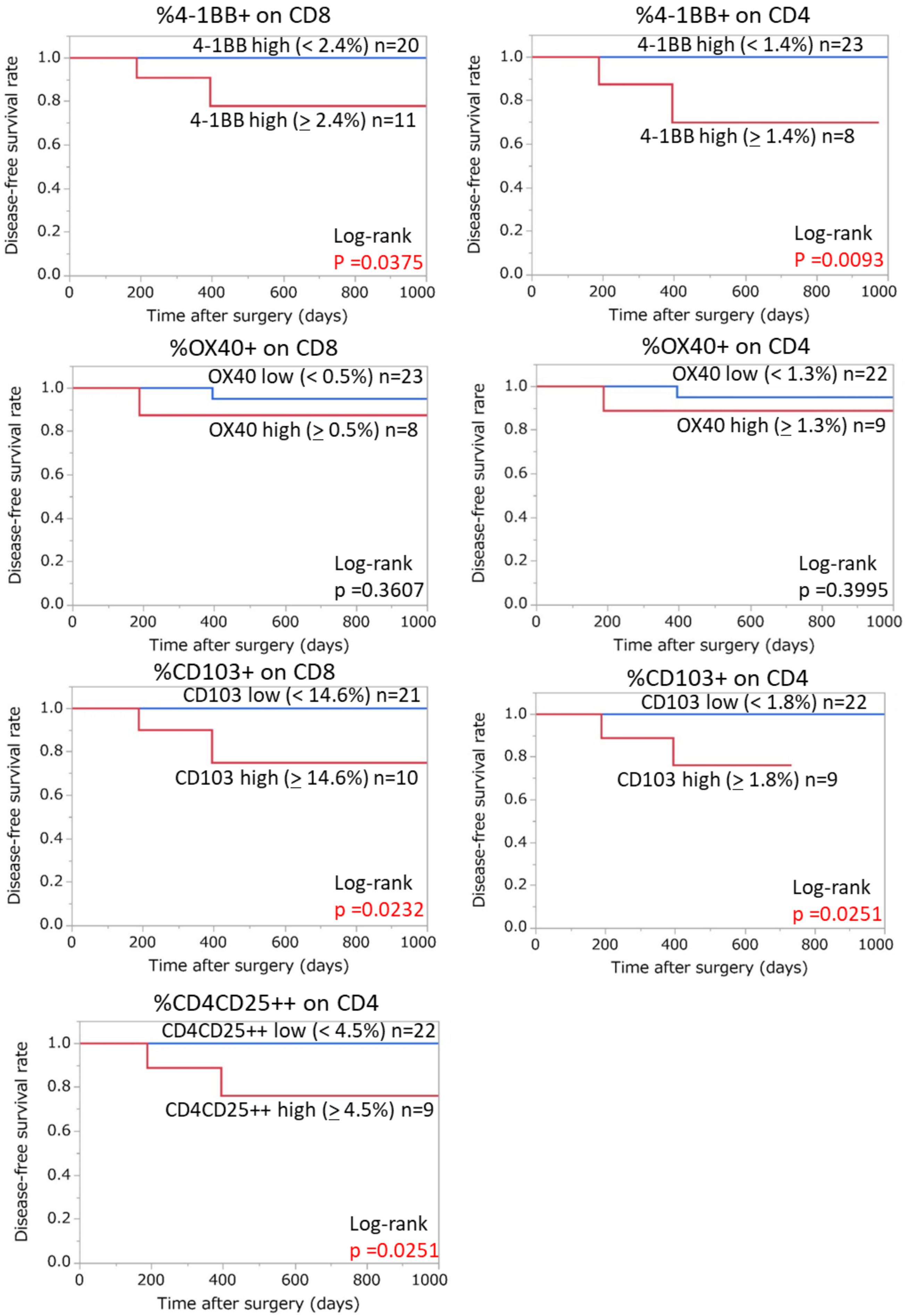
%4-1BB+ on CD8
4-1BB high (< 2.4%) n=20
4-1BB high (≥ 2.4%) n=11
Log-rank
P =0.0375
Disease-free survival rate
Time after surgery (days)
%4-1BB+ on CD4
4-1BB high (< 1.4%) n=23
4-1BB high (≥ 1.4%) n=8
Log-rank
P =0.0093
Disease-free survival rate
Time after surgery (days)
%OX40+ on CD8
OX40 low (< 0.5%) n=23
OX40 high (≥ 0.5%) n=8
Log-rank
p =0.3607
Disease-free survival rate
Time after surgery (days)
%OX40+ on CD4
OX40 low (< 1.3%) n=22
OX40 high (≥ 1.3%) n=9
Log-rank
p =0.3995
Disease-free survival rare
Time after surgery (days)
%CD103+ on CD8
CD103 low (< 14.6%) n=21
CD103 high (≥ 14.6%) n=10
Log-rank
p =0.0232
Disease-free survival rate
Time after surgery (days)
%CD103+ on CD4
CD103 low (< 1.8%) n=22
CD103 high (≥ 1.8%) n=9
Log-rank
p =0.0251
Disease-free survival rate
Time after surgery (days)
%CD4CD25++ on CD4
CD4CD25++ low (< 4.5%) n=22
CD4CD25++ high (≥ 4.5%) n=9
Log-rank
p =0.0251
Disease-free survival rate
Time after surgery (days)

Supplementary Figure S2

Disease-free survival curve of patients with high and low rates of expression for surface molecules on T cells in TET tissues.

The mean rates of expression for surface molecules on T cells in TET tissues were calculated. The disease-free survival curve of patients with high (≥mean) and low (<mean) rates of expression for surface molecules on T cells in TET tissues was shown.

Supplementary Figure S3

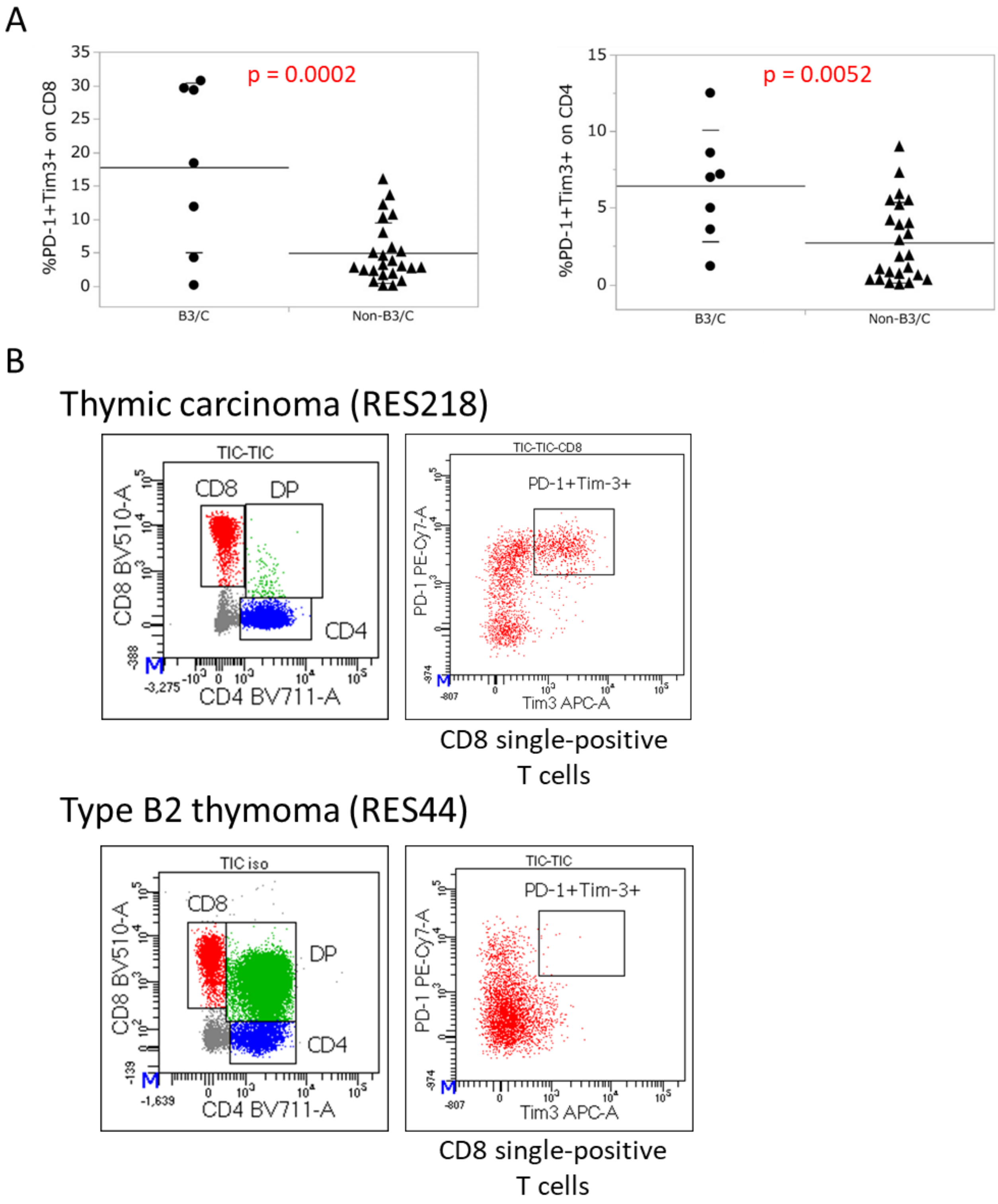

Supplementary Figure S3

Co-expression of Tim-3 and PD-1 on CD4 and CD8 single-positive T cells in B3/C and non-B3/C TET tissues.

A, The percentages of co-expression of Tim-3 and PD-1 on CD8 or CD4 single-positive T cells were analyzed by flow cytometry. B, Representative flow cytometric results.

Supplementary Figure S4

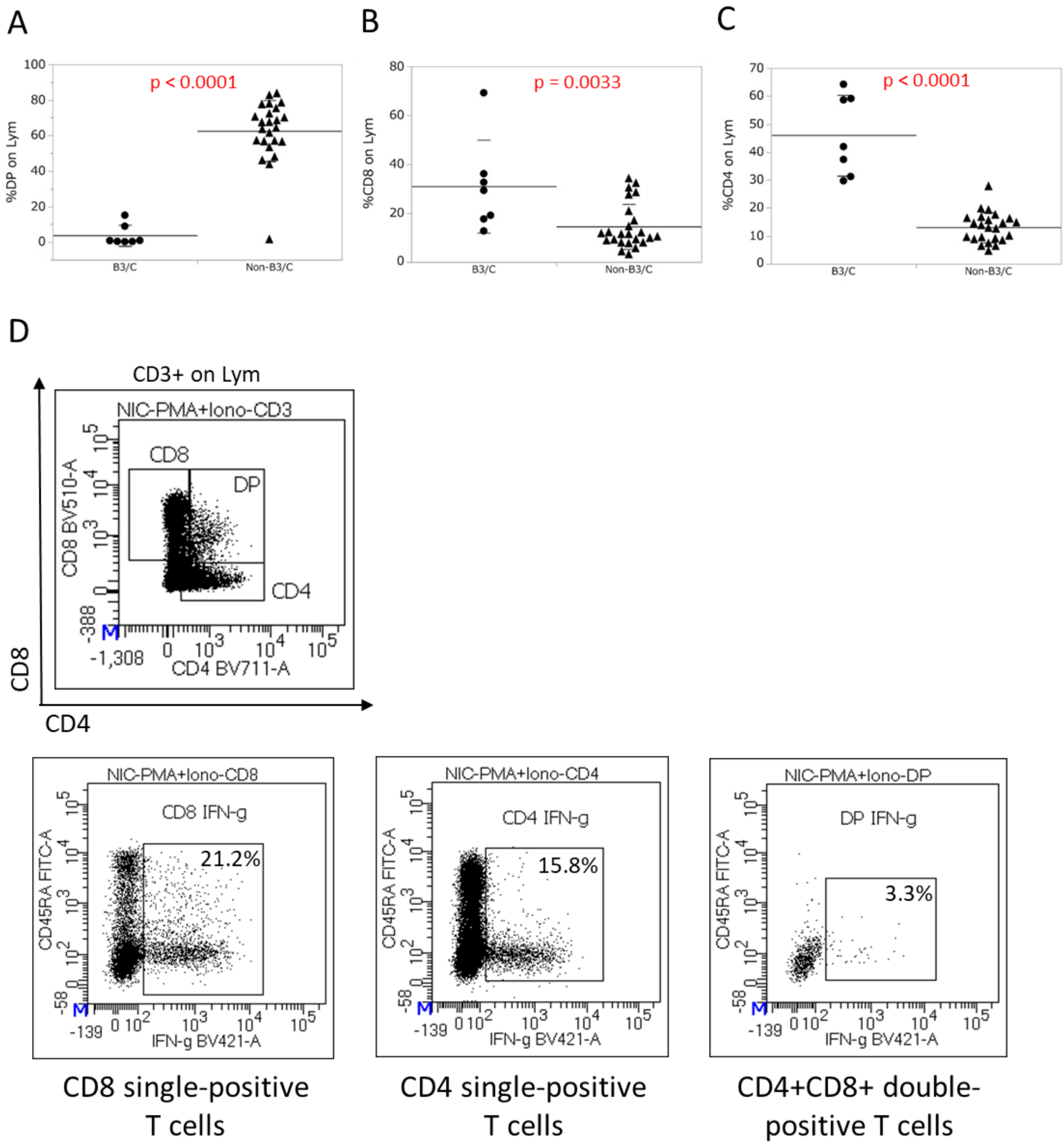

Supplementary Figure S4

CD4 and CD8 single-positive and CD4+CD8+ double-positive T cells in TET tissues.

The proportions of CD4+CD8+ double-positive (**A**), CD8 single-positive (**B**), and CD4 single-positive T cells (**C**) in B3/C and non-B3/C TET tissues were analyzed by flow cytometry. Means with SD were shown. **D**, T cells in normal thymus tissue were stimulated by PMA/ionomycin and analyzed for IFN-γ cytokine production by CD4 and CD8 single-positive and CD4+CD8+ double-positive T cells.

Supplementary Figure S5

PMA + Ionomycin Stimulation Bulk TIC (RES217)

	FITC	PerCP eFluor 710	BV421	BV510	BV605	BV711	Alexa Fluor 700	APC Cy7
TIC lympho	CD45RA	IL-2	IFN-γ	TNF-α	CD8	CD4	CD3	Live/Dead
TIC lympho iso	CD45RA	iso	Iso	CD8	iso	CD4	CD3	Live/Dead

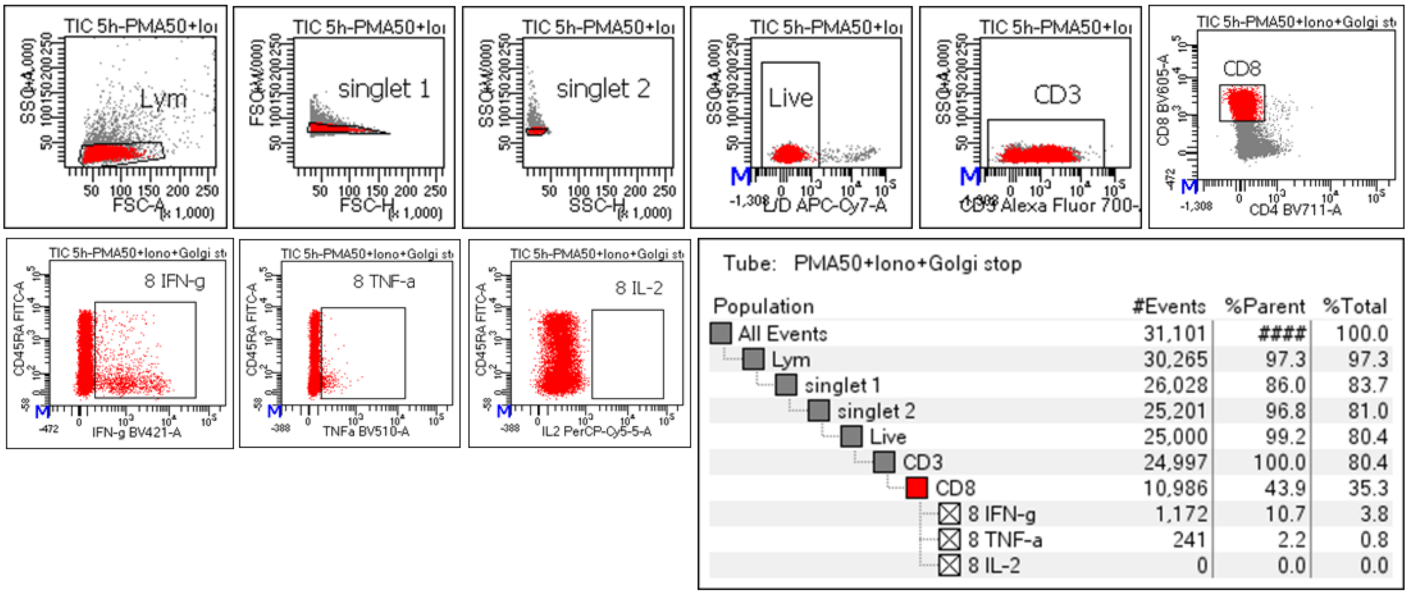

Tube: PMA50+Iono+Golgi stop

Population	#Events	%Parent	%Total
All Events	31,101	####	100.0
Lym	30,265	97.3	97.3
singlet 1	26,028	86.0	83.7
singlet 2	25,201	96.8	81.0
Live	25,000	99.2	80.4
CD3	24,997	100.0	80.4
CD8	10,986	43.9	35.3
8 IFN-g	1,172	10.7	3.8
8 TNF-a	241	2.2	0.8
8 IL-2	0	0.0	0.0

PMA + Ionomycin Stimulation Bulk TIC isotype-control (RES217)

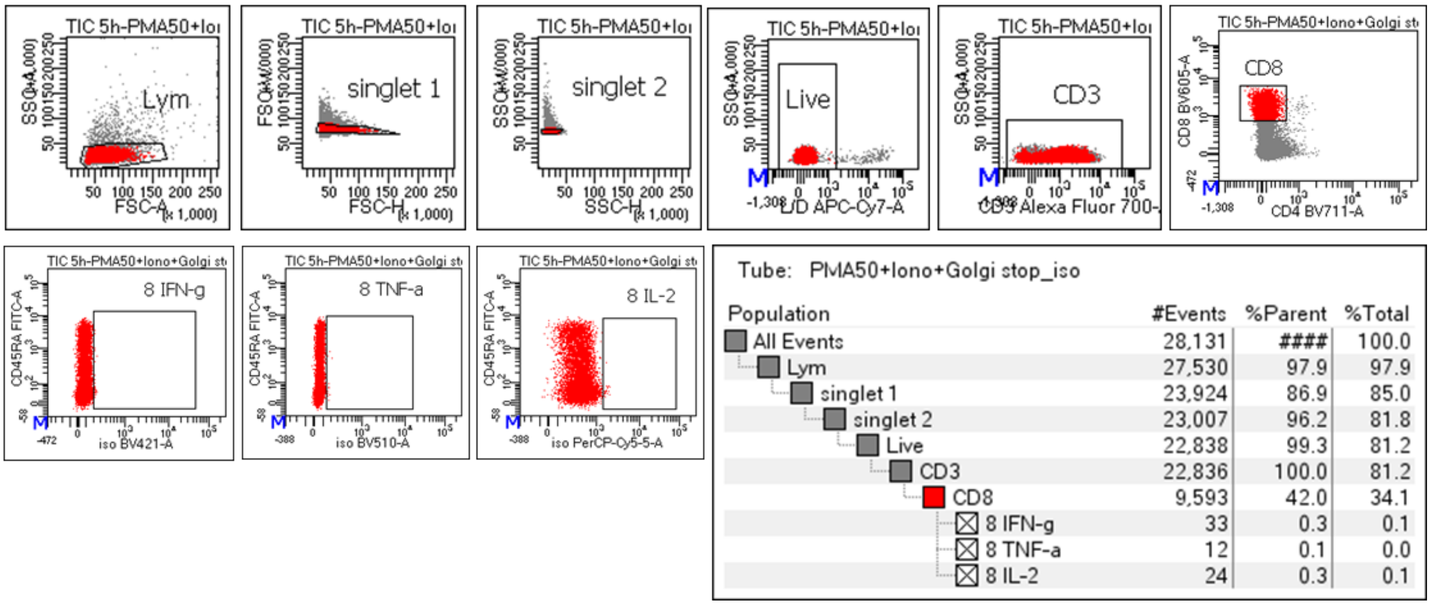

Tube: PMA50+Iono+Golgi stop_iso

Population	#Events	%Parent	%Total
All Events	28,131	####	100.0
Lym	27,530	97.9	97.9
singlet 1	23,924	86.9	85.0
singlet 2	23,007	96.2	81.8
Live	22,838	99.3	81.2
CD3	22,836	100.0	81.2
CD8	9,593	42.0	34.1
8 IFN-g	33	0.3	0.1
8 TNF-a	12	0.1	0.0
8 IL-2	24	0.3	0.1

PMA + Ionomycin Stimulation Sorting CD4 TIC (RES196)

	FITC	PerCP eFluor 710	BV421	BV510	APC	Alexa Fluor 700	APC Cy7
TIC lympho	CD8	IL-2	IFN-γ	TNF-α	CD4	CD3	Live/Dead
TIC lympho iso	CD8	iso	Iso	CD8	CD4	CD3	Live/Dead

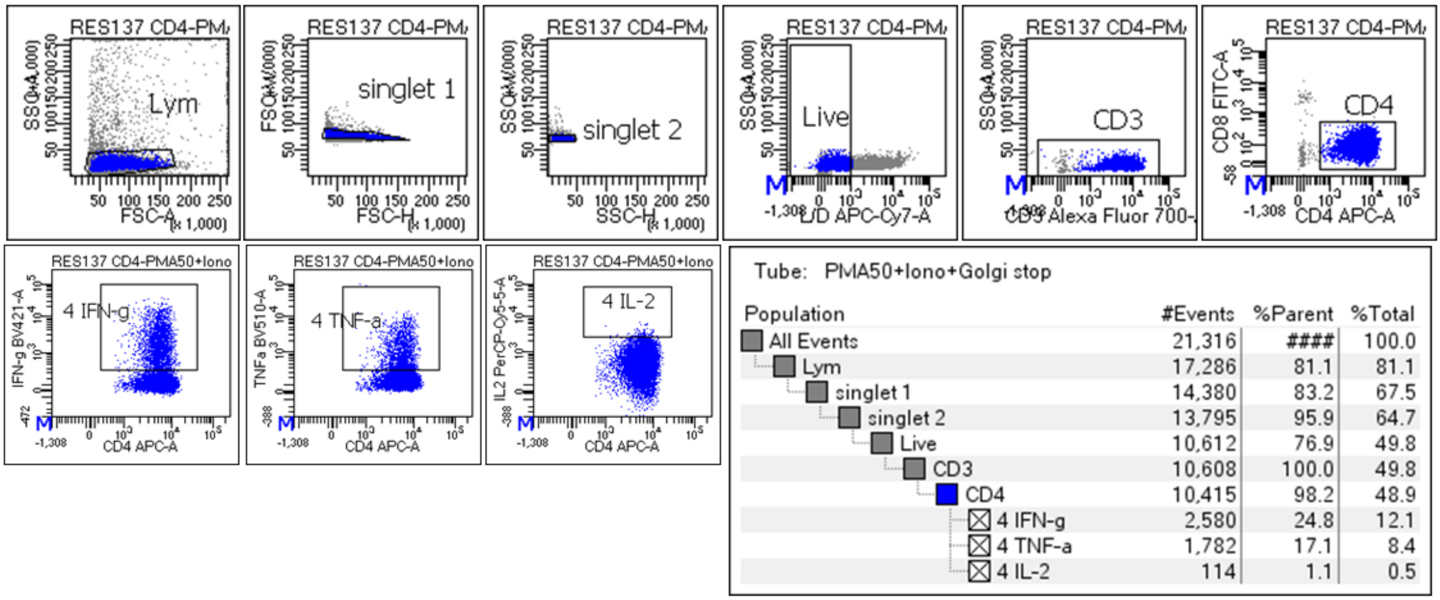

Tube: PMA50+Iono+Golgi stop

Population	#Events	%Parent	%Total
All Events	21,316	####	100.0
Lym	17,286	81.1	81.1
singlet 1	14,380	83.2	67.5
singlet 2	13,795	95.9	64.7
Live	10,612	76.9	49.8
CD3	10,608	100.0	49.8
CD4	10,415	98.2	48.9
4 IFN-g	2,580	24.8	12.1
4 TNF-a	1,782	17.1	8.4
4 IL-2	114	1.1	0.5

PMA + Ionomycin Stimulation Sorting CD4 TIC isotype-control (RES196)

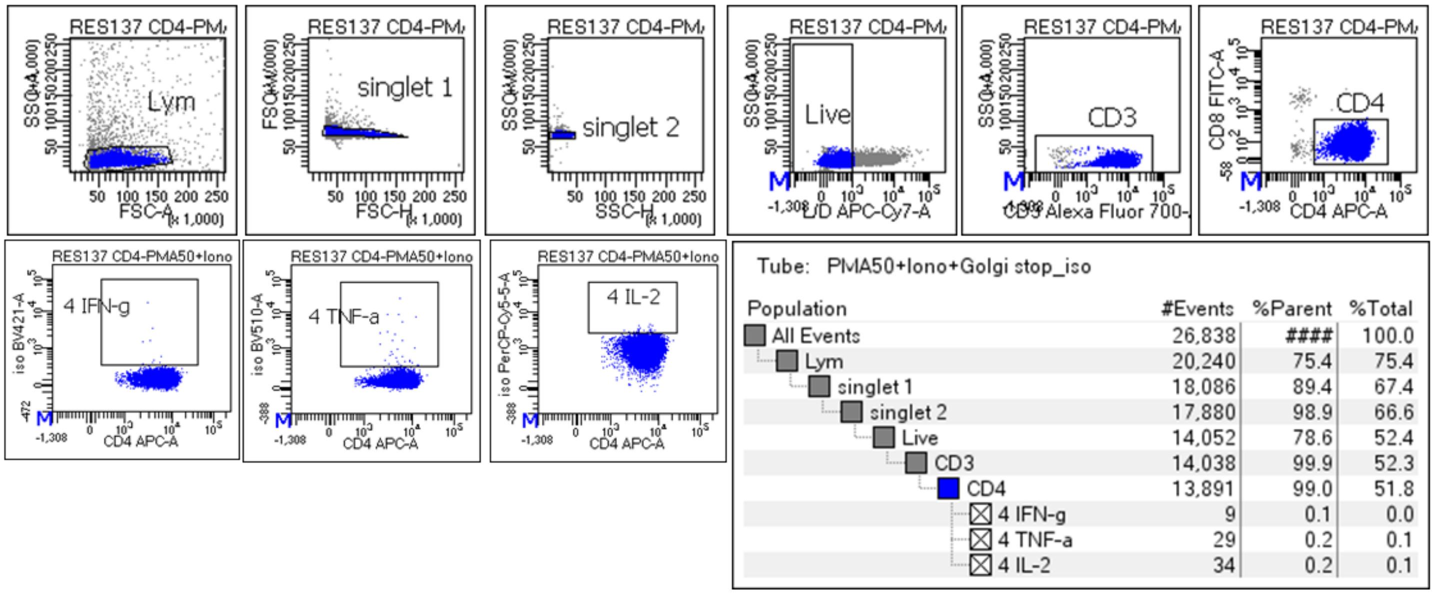

Tube: PMA50+Iono+Golgi stop_iso

Population	#Events	%Parent	%Total
All Events	26,838	####	100.0
Lym	20,240	75.4	75.4
singlet 1	18,086	89.4	67.4
singlet 2	17,880	98.9	66.6
Live	14,052	78.6	52.4
CD3	14,038	99.9	52.3
CD4	13,891	99.0	51.8
4 IFN-g	9	0.1	0.0
4 TNF-a	29	0.2	0.1
4 IL-2	34	0.2	0.1

Supplementary Figure S5

Gating strategy of the FACS analysis for intracellular cytokine staining.

The gating strategy of intracellular cytokine staining was shown using BD LSRFortessa with FACSDiva software.

Supplementary Figure S6

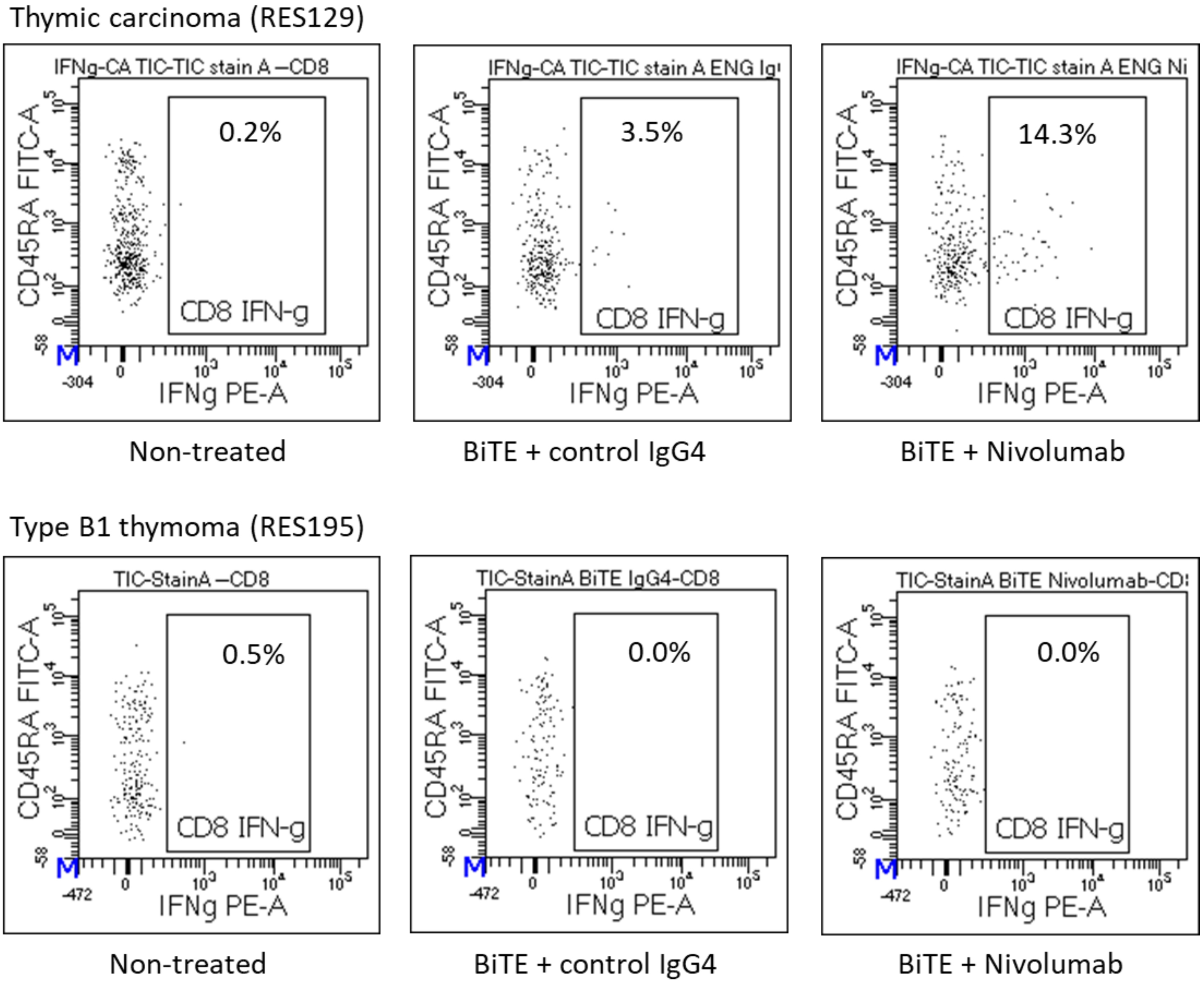

Supplementary Figure S6

IFN-γ secretion assay for TET tissues.

Representative flow cytometric results for the IFN-γ secretion assay in Fig. 4B were shown for thymic carcinoma (RES129) and type B1 thymoma (RES195) patients.

Supplementary Figure S7

A

Type B2 Thymoma (RES118)

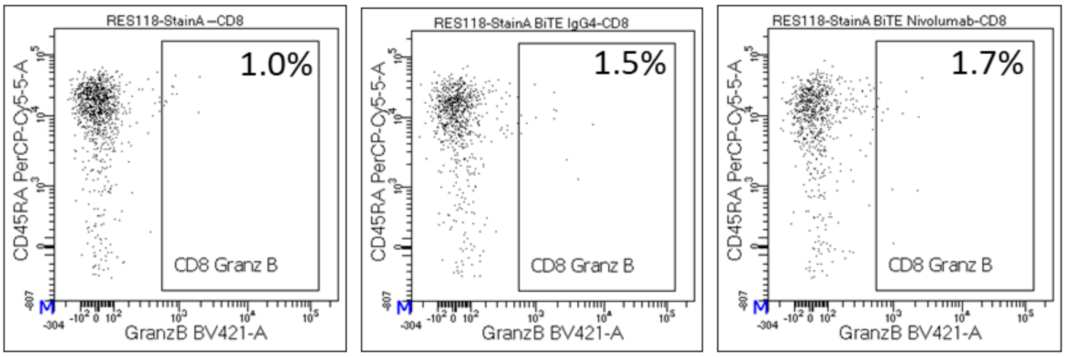

Non-treated | BiTE + control IgG4 | BiTE + Nivolumab

Thymic carcinoma

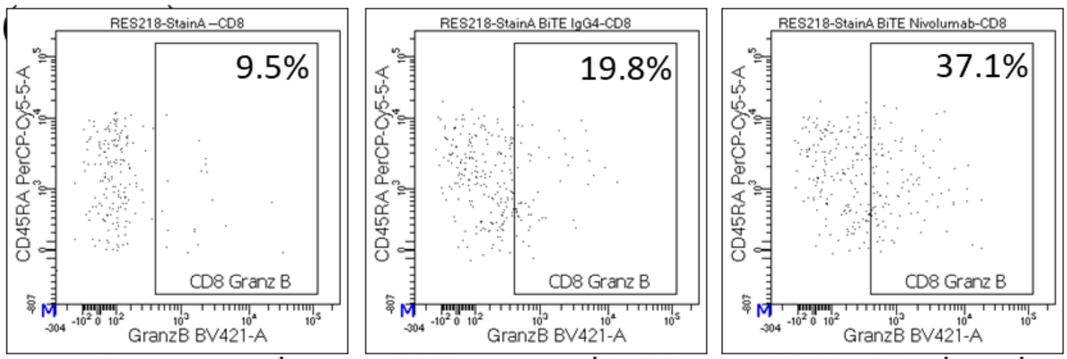

Non-treated | BiTE + control IgG4 | BiTE + Nivolumab

B

Type B2 Thymoma (RES118)

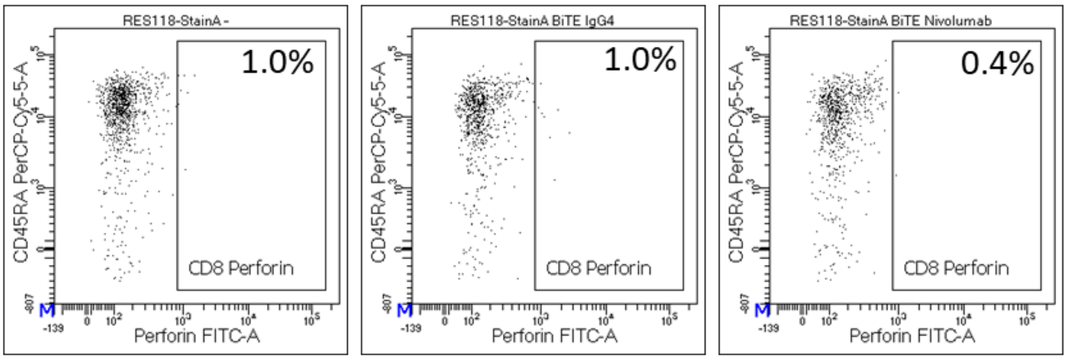

Non-treated | BiTE + control IgG4 | BiTE + Nivolumab

Thymic carcinoma (RES218)

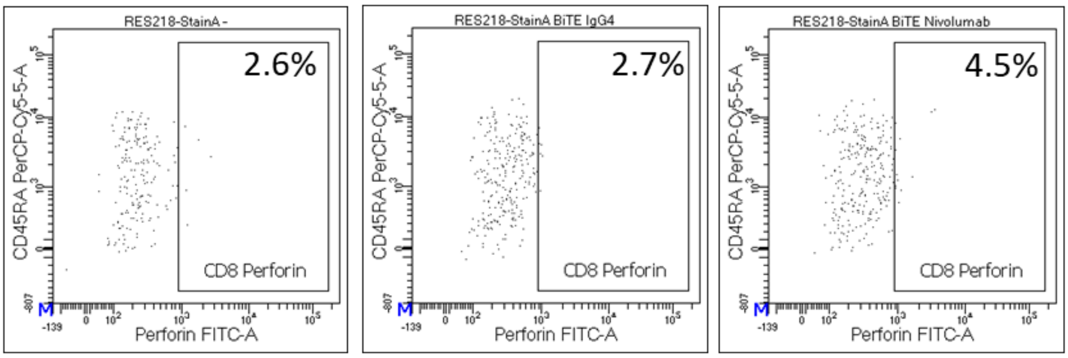

Non-treated | BiTE + control IgG4 | BiTE + Nivolumab

Supplementary Figure S7

Granzyme B and perforin expression in CD8 single-positive T cells in B3/C and non-B3/C TETs.

Cryopreserved cells isolated from TET tissues were thawed and co-cultured with U251 cells, EphA2/CD3 BiTE, and nivolumab. After a 48-hour co-culture, the intracellular staining of granzyme B (A) and perforin (B) was analyzed.

Supplementary Figure S8

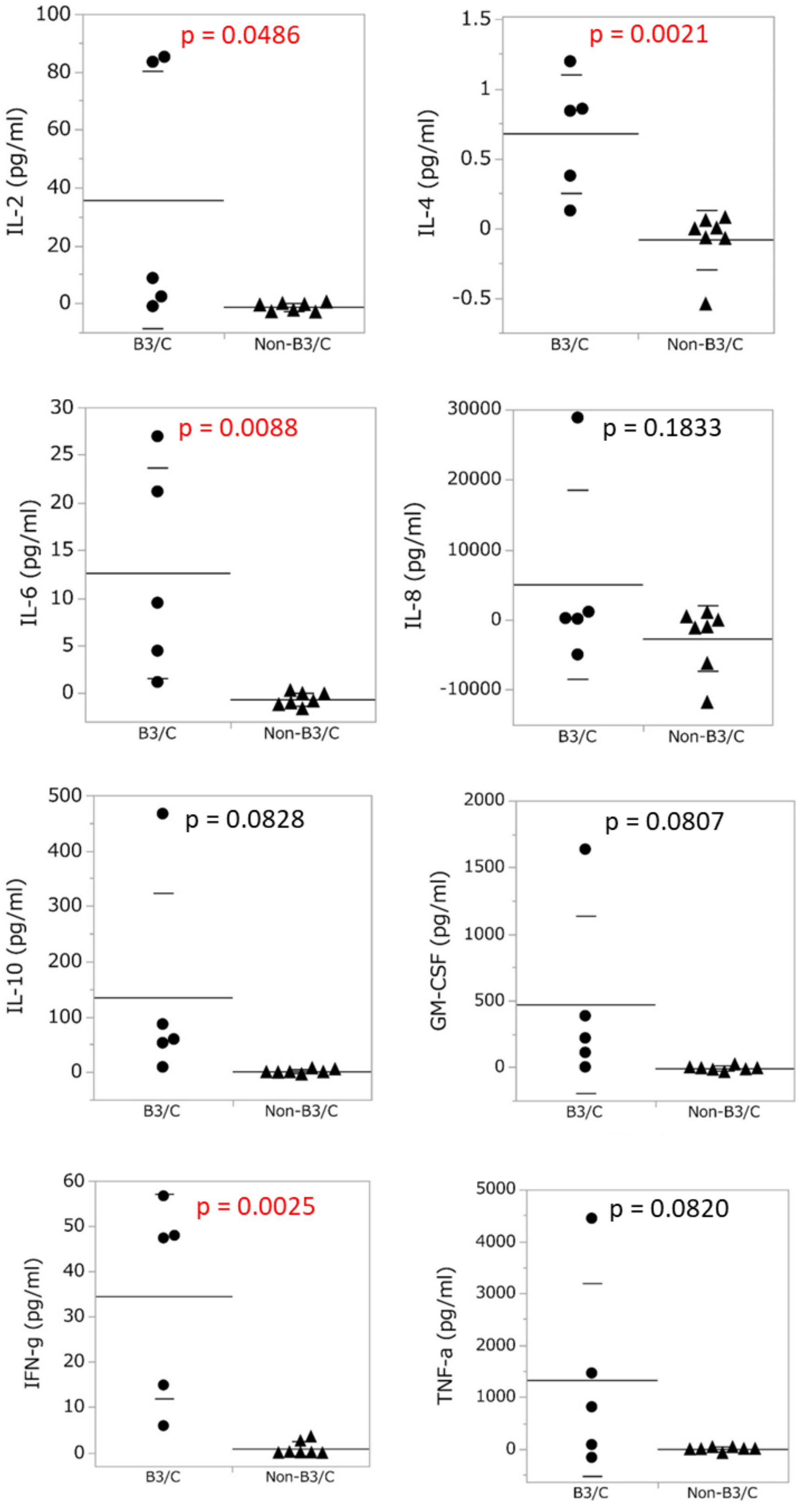

Supplementary Figure S8

Multiplex cytokine assay for TET tissues.

Cytokine concentrations in co-cultured supernatants in Fig. 4 were analyzed by the multiplex cytokine assay. Means with SD were shown.

Supplementary Figure S9

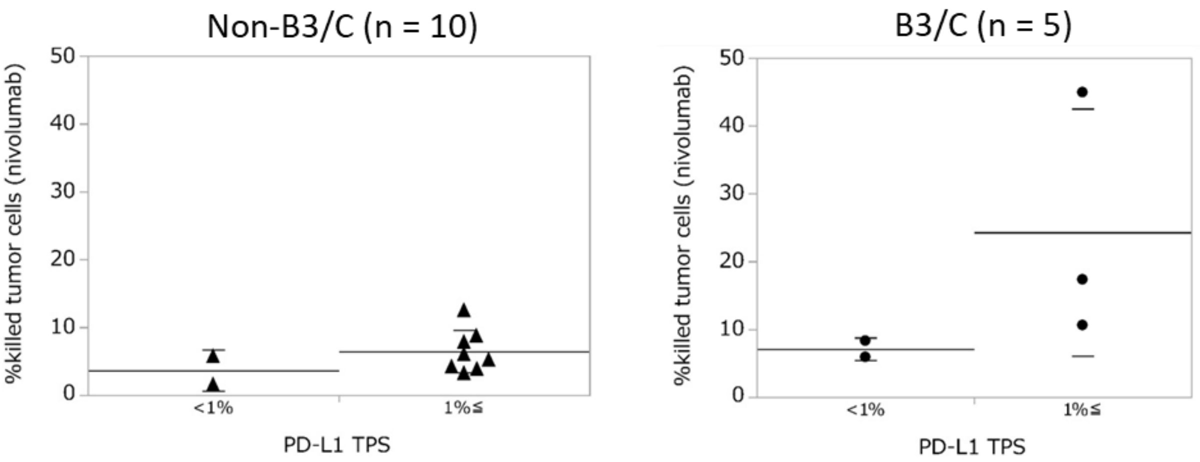

Supplementary Figure S9

PD-L1 expression on tumor cells in TET tissues.

PD-L1 expression on tumor cells was analyzed by immunohistochemistry using anti-PD-L1 antibodies (clone E1L3N). The ratio of PD-L1 expression in tumor cells (tumor proportion score, TPS) was analyzed. The effects of nivolumab on T cells in TET tissues were analyzed by T-cell cytotoxicity using BiTE with nivolumab. The effects of nivolumab on T-cell cytotoxicity in TPS high (≥1%) and low (<1%) groups were evaluated. Means with SD were shown.

Supplementary Figure S10

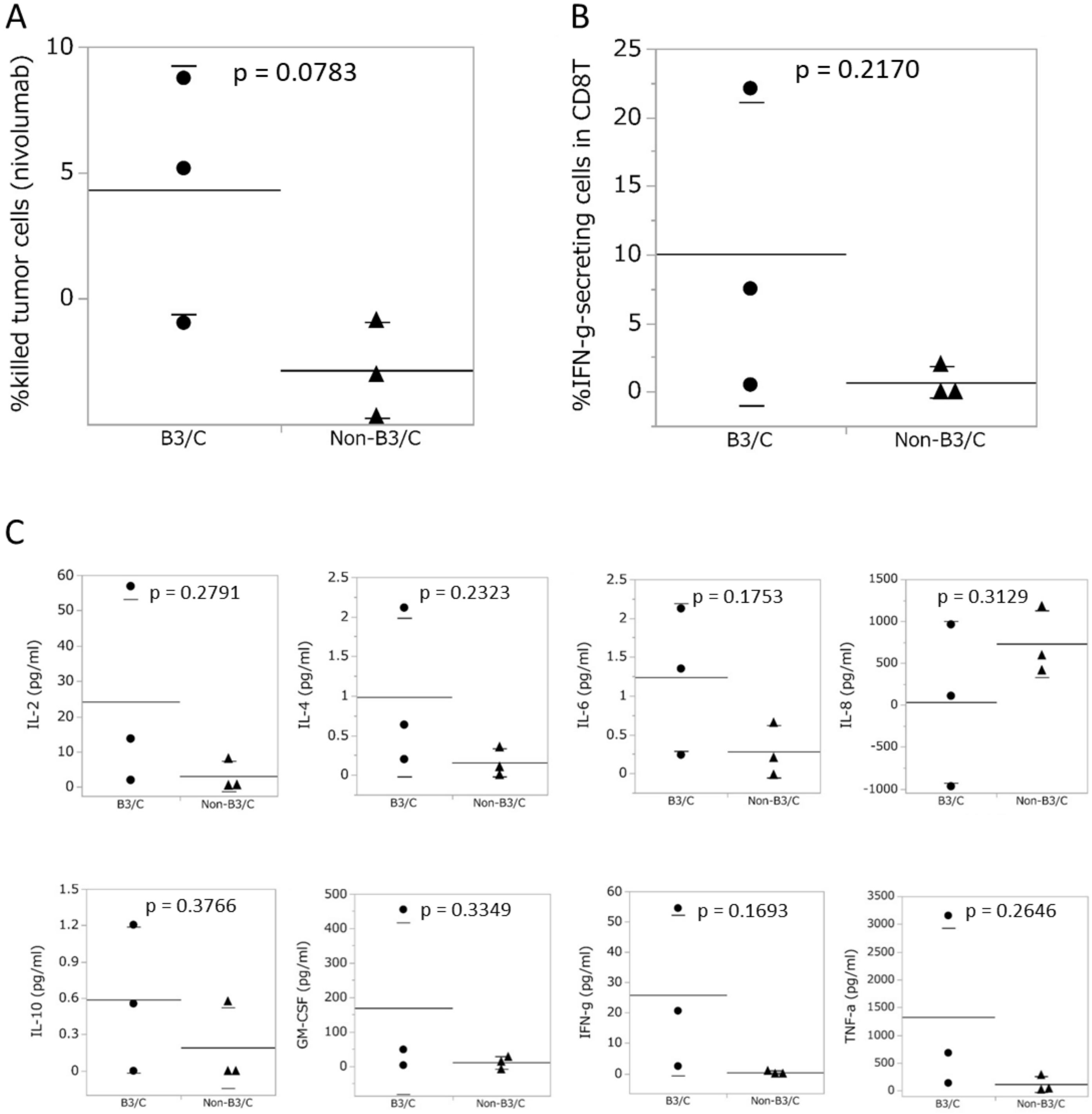

Supplementary Figure S10

Effects of nivolumab on T-cell cytotoxicity for purified CD8 single-positive T cells from B3/C and non-B3/C TET tissues.

A, The effects of nivolumab on purified CD8 single-positive T cells from six TET tissues were analyzed by T-cell cytotoxicity using BiTE with nivolumab. B, IFN-γ secretion from CD8 single-positive T cells was also evaluated. C, Cytokine concentrations of co-cultured supernatants were analyzed by a multiplex cytokine assay. Means with SD were shown.

Supplementary Table S1.

Univariate analysis of factors associated with T-cell profiles (Hot and Cold clusters)

	Hot	Cold	p value
Age (<62/≥62)	2/5	12/12	0.3162
Sex (Male/Female)	4/3	6/18	0.1094
Myasthenia gravis (Present/Absent)	0/7	4/20	0.2471
Tumor size (<25.8 cm^3/≥25.8 cm^3)	4/3	11/13	0.5983
Masaoka classification (I/II+III+V)	2/5	13/11	0.2331
SUVmax (<4.1/≥4.1)	0/6	12/6	0.0047
Histology (B3-C/non B3-C)	7/0	0/24	<0.0001

Supplementary Table S2.

PD-L1 expression on tumor cells in TETs

	PD-L1 expression	
	<1%	≧1%
B3/C	2	4
Non-B3/C	8	15
